# Enzyme‐Inspired Hydrogen‐Bonded Organic Frameworks for Synergistic Capture, Detection, and Degradation of Nerve Agent Simulants

**DOI:** 10.1002/advs.202519971

**Published:** 2025-11-23

**Authors:** Jiabao Liu, Guanglai Mo, Xiangyu Gao, Yingjia Deng, Yijin Wang, Qingyu Niu, Yujie Lei, Bin Fei, Joanne Yip, Zhaozhen Zhang, Jie Wu, Yunbo Bi, Kaikai Ma, Zhiqiang Li, Peng Li

**Affiliations:** ^1^ School of Chemical Engineering and Technology Hebei University of Technology Xiping Dao 5340, Beichen District Tianjin 300401 China; ^2^ State Key Laboratory of Porous Materials for Separation and Conversion Shanghai Key Laboratory of Molecular Catalysis and Innovative Materials Department of Chemistry College of Smart Materials and Future Energy Fudan University 2005 Songhu Road Shanghai 200438 China; ^3^ School of Fashion and Textiles The Hong Kong Polytechnic University Hung Hom Kowloon Hong Kong SAR 999077 China; ^4^ Research Center of Green Catalysis College of Chemistry Zhengzhou University Zhengzhou 450001 China

**Keywords:** hydrogen‐bonded organic frameworks, nerve agent simulants, instantaneous capture, selective recognition, spontaneous degradation

## Abstract

Nerve agents (NAs), a class of highly toxic organophosphorus (OP) compounds, pose a significant threat to global security. The development of integrated protective materials that can simultaneously capture airborne OPs, detect, and degrade them remains a formidable challenge. Inspired by lipase's specific binding to OPs, a biomimetic hydrogen‐bonded organic framework (HOF) is developed, FDU‐HOF‐5. It achieves highly efficient and selective adsorption of a sarin simulant, diethyl chlorophosphate (DCP), by combining size exclusion with molecular recognition via specific N─P bond formation, effectively distinguishing DCP from various OP analogues. Upon adsorption, the material responds within 5 s, enabling bimodal identification via a visual color change (yellow to red) and a fluorescence signal (99.7% quenching, 75 nm redshift). The adsorbed DCP is then self‐drivenly hydrolyzed into non‐toxic products by environmental moisture, achieving 91.5% degradation efficiency. Utilizing the solution processability of HOFs, functional textiles that show an immediate color change upon DCP exposure are produced. This work establishes a rational design strategy for multifunctional HOF systems that synergistically integrate capture, detection, and degradation for effective threat mitigation.

## Graphical abstract

FDU‐HOF‐5, an enzyme‐inspired hydrogen‐bonded framework, enables targeted capture, real‐time visual alert, and detoxification of nerve agent simulants through biomimetic sites and multivalent interactions. Integrable into protective gear, it pioneers on‐site threat monitoring strategies for persistent neurotoxic hazards, advancing smart, responsive materials in public safety.

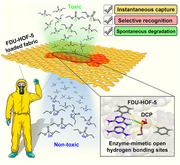

## 1 Introduction

**Scheme 1:**
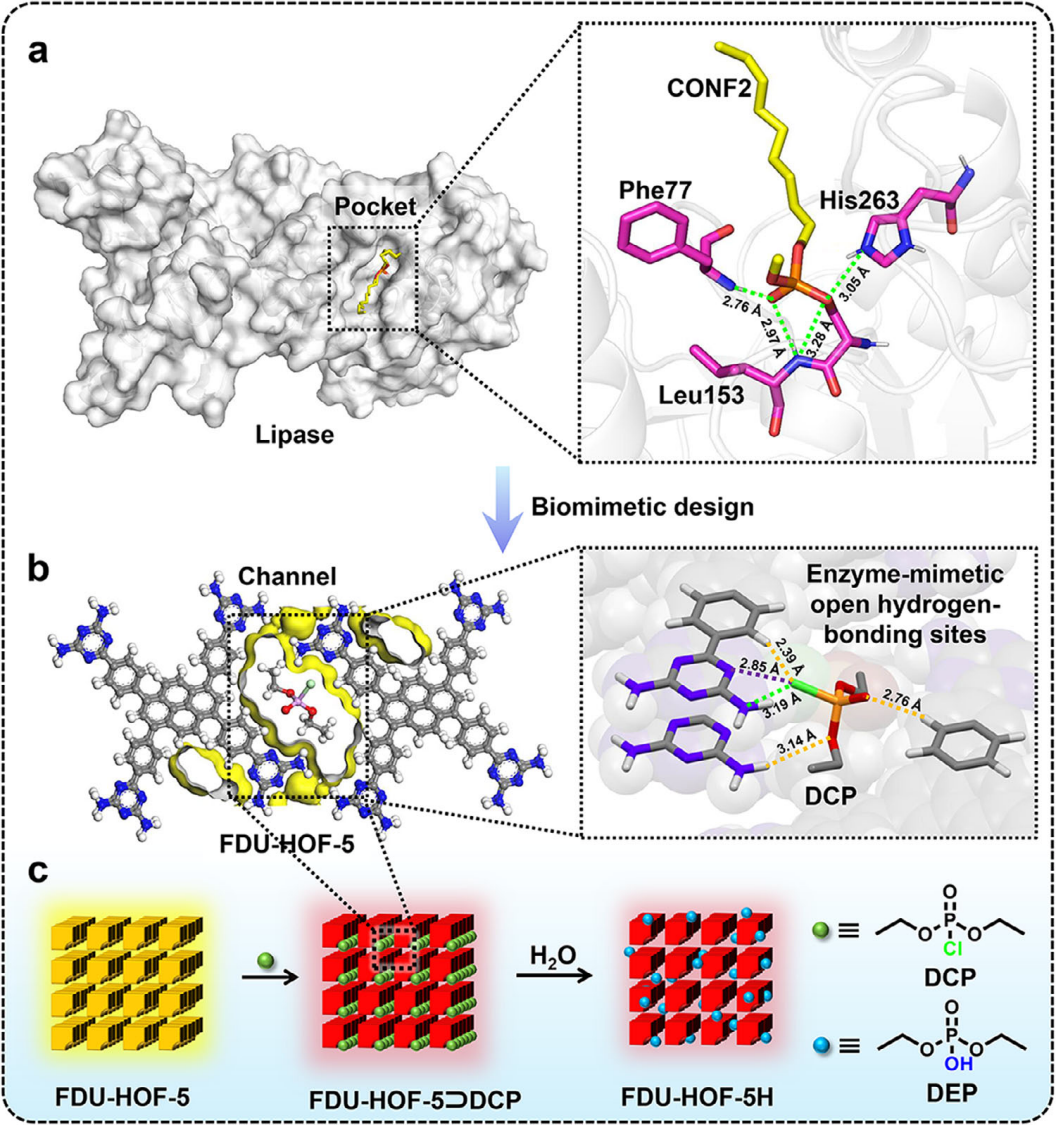
Schematic diagram of the trifunctional capture, detection, and degradation of sarin simulants by FDU‐HOF‐5, inspired by lipase. a) The “pocket” of lipase and the hydrogen‐bond interaction between the amino acid residues in the “pocket” and C11 alkyl phosphonate (CONF2); b) The interaction between the FDU‐HOF‐5 biomimetic channel and its open sites with DCP, the green dotted lines represent hydrogen bonds, the purple dotted lines represent halogen bonds, and the orange dotted lines represent weak van der Waals forces; c) The FDU‐HOF‐5 with integrated functions of capture, detection and degradation of DCP.

Nerve agents (NAs) are highly toxic organophosphorus (OP) compounds that induce lethal systemic poisoning through minimal exposure. Due to their extreme volatility and rapid permeation via the skin or respiratory routes, they can cause fatal respiratory failure within minutes in an emergency, posing a severe risk to public safety.^[^ ^1^, ^2^ ^]^ Even sublethal doses rapidly disrupt neurotransmission, while delayed diagnosis and narrow antidote windows exacerbate threats from accidental leaks or malicious use, perpetuating global security and health crises.^[^ ^3^, ^4^, ^5^ ^]^ To counter these threats, the development of advanced multifunctional materials capable of directly capturing, rapidly colorimetric detecting, and spontaneously degrading low‐concentration toxic gases in the air has emerged as a critical research frontier for the establishment of next‐generation wearable, portable, and smart protective gears against chemical hazards.^[^ ^6^, ^7^ ^]^ Previous studies have extensively investigated single‐functional materials for the adsorption, degradation, or detection of NA. For instance, porous materials such as carbonaceous nanoporous materials, metal–organic frameworks (MOFs), boron nitride, and zeolites have been explicitly explored for NA capture.^[^ ^8^, ^9^, ^10^, ^11^ ^]^ By strategically engineering metallic Lewis acidic sites or reactive nucleophilic motifs, MOFs,^[^ ^12^ ^]^ metal oxide nanoparticles,^[^ ^13^ ^]^ and reactive polymers exhibit programmable hydrolytic NA‐degradation functionalities.^[^ ^14^ ^]^ To realize portable detection of NA, various fluorescent small‐molecule probes and their composites have been designed to enable portable, visualization‐based detection;^[^ ^15^, ^16^, ^17^, ^18^ ^]^ Although the tunability of MOF‐based materials has enabled the design of systems capable of simultaneously adsorbing and degrading NA,^[^ ^19^, ^20^, ^21^, ^22^ ^]^ integrating highly specific and sensitive detection functions without compromising the core adsorption‐degradation efficacy remains a critical scientific challenge. A promising approach to addressing this bottleneck is to construct biomimetic porous materials that emulate the specific recognition mechanism of enzymes,^[^ ^23^ ^]^ as exemplified by lipases that identify target OPs through multiple hydrogen‐bond interactions with analytes within their precisely tailored 3D active “pockets” (**Scheme** **1**a).^[^ ^24^ ^]^

As a novel class of crystalline porous materials, hydrogen‐bonded organic frameworks (HOFs), self‐assembled through weak intermolecular forces (hydrogen bonding, *π–π* stacking, van der Waals interactions),^[^ ^25^, ^26^, ^27^ ^]^ have demonstrated multifunctional potential across adsorption, separation, sensing, and catalytic applications.^[^ ^28^, ^29^, ^30^, ^31^ ^]^ Mirroring the design principles of MOFs, the modular architecture of HOFs empowers systematic and synergistic control over porosity, surface chemistry, and optoelectronic properties through the precision engineering of hydrogen‐bonding functionalized organic tectons.^[^ ^32^, ^33^, ^34^ ^]^ Most importantly, by judicious choice of building tectons, the nanopores of HOFs with abundant open hydrogen‐bonding sites (OHSs) can be rationally designed to mimic the structures of lipase enzymes. Among numerous potential hydrogen‐bonding terminal units, we have identified diaminotriazine (DAT) as the ideal candidate for this purpose due to its three key attributes: 1) DAT is a nitrogen‐rich heterocycle, providing abundant OHSs for multi‐point weak interactions with OPs; 2) the resulting protonated DAT species enhances the intramolecular charge transfer (ICT) effect in the donor‐π‐acceptor (D‐π‐A) architecture, leading to pronounced optical signal changes;^[^ ^35^ ^]^ 3) the Brønsted basicity of N atoms on DAT could facilitate the OP hydrolysis.^[^ ^36^ ^]^

Inspired by enzyme‐mediated selective recognition, we synthesized FDU‐HOF‐5 through the modular self‐assembly of pyrene‐DAT derivatives (Py‐4DAT). Its 1D micropores (≈12.6 Å × 7.1 Å) spatially match the dimensions of diethyl chlorophosphate (DCP, a sarin simulant), enabling the selective capture of this compound via hydrogen/halogen bonding and van der Waals interactions that mimic the “pockets” found in enzymes (Scheme 1b). Upon adsorption, simultaneous ICT activation induces a rapid (≈5 s) visual colorimetric change from yellow to red, 99.7% fluorescence quenching, and a 75 nm spectral redshift, achieving a detection limit of 31 ppb. The adsorbed DCP is further hydrolyzed to the non‐toxic diethyl phosphate (DEP) under ambient humidity with 91.5% efficiency. When integrated onto fabrics, the platform demonstrates real‐time field applicability. By combining enzyme‐inspired capture, detection, and degradation within a single material (Scheme 1c), this work offers an innovative on‐demand solution for mitigating chemical threats.

## 2 Results and Discussion

### 2.1 Synthesis and Characterizations of FDU‐HOF‐5

**Figure 1:**
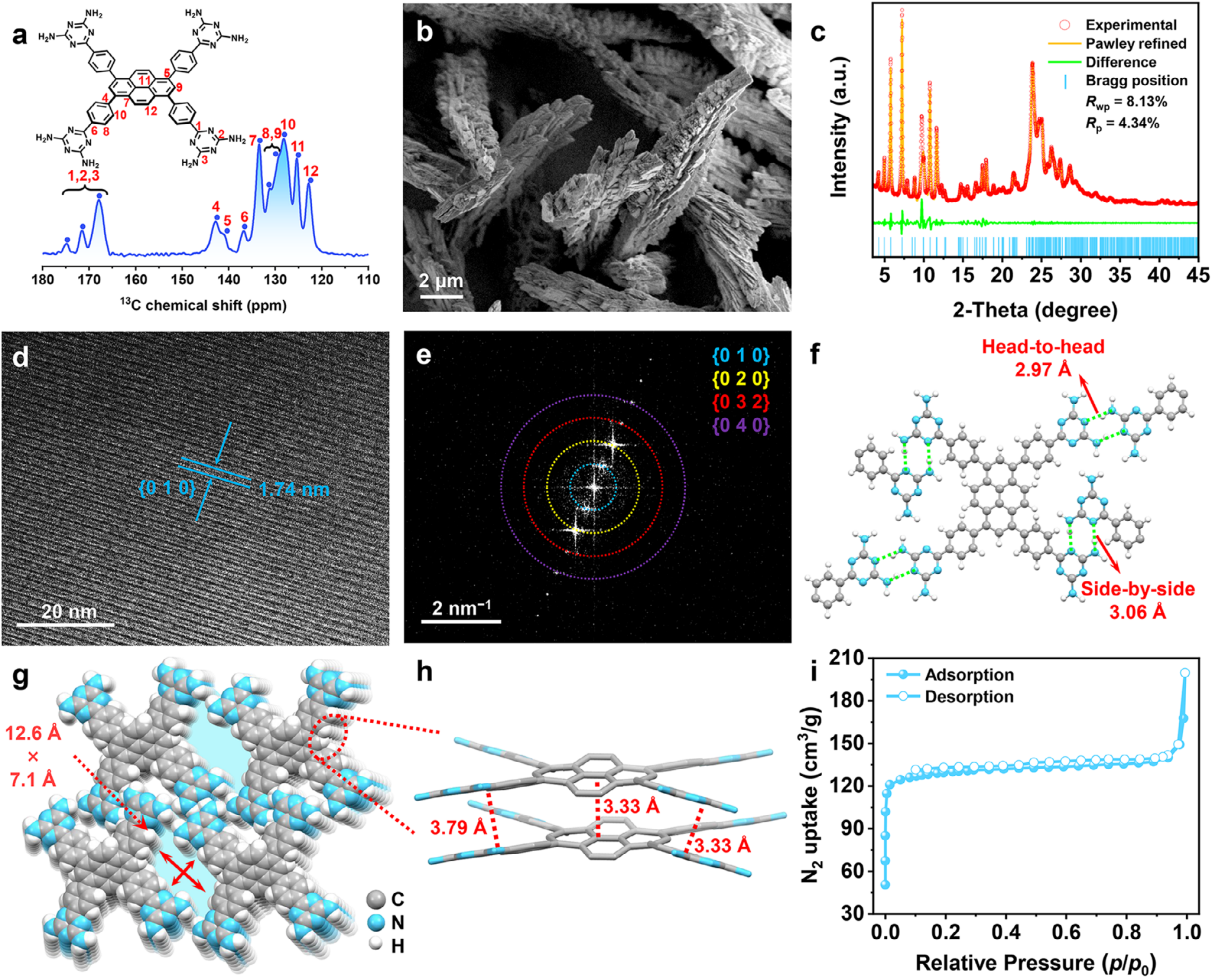
The structure and characterization of FDU‐HOF‐5. a) ^13^C ssNMR spectra and structure diagram of FDU‐HOF‐5; b) The SEM image of FDU‐HOF‐5; c) PXRD patterns and Rietveld refinement results for FDU‐HOF‐5; d) The high‐resolution cryo‐TEM image of FDU‐HOF‐5; e) The FFT image of FDU‐HOF‐5; f) Head‐to‐head hydrogen bonds and side‐by‐side hydrogen bonds in FDU‐HOF‐5; g) 1D channels formed by stacking; h) *π–π* stacking in FDU‐HOF‐5; i) N_2_ adsorption‐desorption isotherms of FDU‐HOF‐5 at 77 K.

The COOH‐modified pyrene‐based molecule H_4_TBAPy has been employed to construct HOF via complementary hydrogen bonds, forming a 2D layered stacking structure denoted as PFC‐1 (HOF‐101).^[^ ^25^, ^26^ ^]^ This material showcases excellent shape‐matched *π–π* stacking interactions and exhibits remarkable stability. By substituting the COOH in H_4_TBAPy with DAT, a novel building block, 6,6′,6′“,6′”'‐(pyrene‐1,3,6,8‐tetrayltetrakis(benzene‐4,1‐diyl))tetrakis(1,3,5‐triazine‐2,4‐diamine) (Py‐4DAT), featuring a D‐π‐A structure (Figure S1, Supporting Information), was synthesized according to the reported method with slight modification (Figure S2, Supporting Information) and characterized by ^1^H NMR (Figure S3, Supporting Information), ^13^C NMR (Figure S4, Supporting Information), and high‐resolution mass spectrometry (Figure S5, Supporting Information).^[^ ^37^, ^38^, ^39^ ^]^ Yellow FDU‐HOF‐5 was rapidly and scalably prepared by pouring acetone into a dimethyl sulfoxide solution of Py‐4DAT (≈30 mg mL^−1^). ^13^C solid‐state NMR (ssNMR, **Figure** **1**a) and powder X‐ray diffraction (PXRD, Figure S6, Supporting Information) results indicate that FDU‐HOF‐5 has a highly ordered crystal structure.^[^ ^40^ ^]^ Scanning electron microscopy (SEM) images (Figure 1b) further reveal that it presents an irregular block‐like microstructure. However, despite numerous attempts, single‐crystal samples suitable for single‐crystal X‐ray diffraction analysis have not been obtained.

The structure model of FDU‐HOF‐5 was constructed through theoretical simulation using the Materials Studio software package with a space group of *P* $\bar{1}$.^[^ ^41^ ^]^ The unit cell parameters are *a* = 3.7986 Å, *b* = 18.1785 Å, *c* = 21.2232 Å, *α* = 77.1783°, *β* = 90.6793°, and *γ* = 92.7270°. Furthermore, the Pawley refinement of the experimental PXRD pattern (Figure S6, Supporting Information) was conducted based on the simulated unit cell parameters,^[^ ^42^ ^]^ producing a reliable fitting factor and low residual values (*R*_p_ = 4.34%, *R*_wp_ = 8.13%, Figure 1c, Table S1, Supporting Information). The accuracy of the refined crystal structure was further verified by high‐resolution cryo‐transmission electron microscopy (cryo‐TEM). Lattice fringes were clearly observed (Figure 1d), and the corresponding fast Fourier transform (FFT) pattern (Figure 1e) revealed distinct diffraction spots that were indexed to crystal planes such as {010}, {020}, {032}, and {040}. The measured interplanar spacing for the {010} family was 1.74 nm (Figure 1d; Figures S7 and S8, Supporting Information), which is in excellent agreement with the theoretical value of 1.77 nm calculated from the PXRD‐refined unit cell (Figure S9, Supporting Information), thereby corroborating the structural model.

In the FDU‐HOF‐5, the Py‐4DAT molecules are connected by head‐to‐head hydrogen bonds (2.97 Å) and side‐by‐side hydrogen bonds (3.06 Å) to form a planar network (Figure 1f). Adjacent layers are stacked via strong *π–π* interactions (Figure 1g,h) to generate a 2D layered stacking structure with a *sql* topology (Figure S10, Supporting Information). This material features open 1D quadrilateral channels with approximate dimensions of 7.1 Å × 12.6 Å when considering the van der Waals radius (Figure 1g). PLATON calculations reveal a theoretical solvent‐accessible void volume of ≈24.9% for FDU‐HOF‐5 (Figure S11, Supporting Information).^[^ ^43^ ^]^ The presence of these permanent pores was confirmed experimentally by N_2_ adsorption measurements at 77 K (Figure 1i). The resulting Type I isotherm and a Brunauer–Emmett–Teller surface area of 395.6 m^2^ g^−1^ are characteristic of a microporous material. Analysis of the pore size distribution using the Horvath–Kawazoe method showed a maximum at 10.2 Å (Figure S12, Supporting Information), which is consistent with the dimensions predicted from the crystal structure.

The material also exhibited excellent stability, a crucial property for practical applications. It maintained its crystallinity up to 200 °C (Figure S13, Supporting Information) and showed no significant weight loss below 208 °C according to thermogravimetric analysis (Figure S14, Supporting Information). Furthermore, FDU‐HOF‐5 demonstrated remarkable chemical stability, retaining its crystalline structure after immersion in various common laboratory solvents and even a 10 m NaOH aqueous solution for 2 days (Figure S15, Supporting Information). This combination of thermal and chemical stability provides a solid foundation for the use of FDU‐HOF‐5 in applications such as the capture and detection of DCP.

### 2.2 Adsorption of OPs by FDU‐HOF‐5

**Figure 2:**
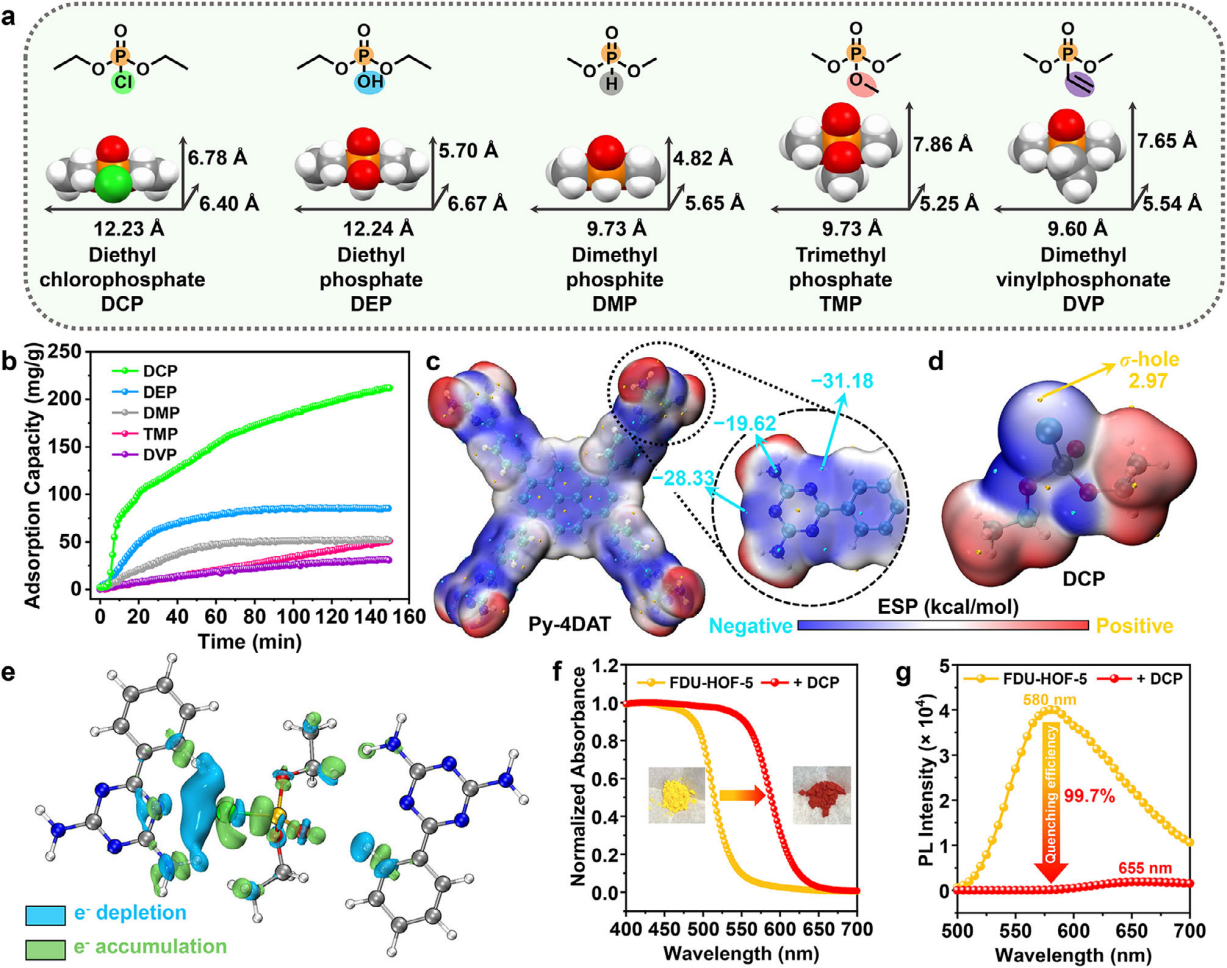
Adsorption tests of FDU‐HOF‐5. a) The structures and molecular dimensions of DCP, DEP, DMP, TMP, and DVP; b) The DVS adsorption curves of FDU‐HOF‐5 for DCP, DEP, DMP, TMP, and DVP at 298 K; The ESP images of c) Py‐4DAT and d) DCP, yellow spheres correspond to the maxima of electrostatic potential and cyan spheres correspond to the minima of electrostatic potential; e) The electron density difference map of FDU‐HOF‐5⊃DCP; f) The UV–vis absorption spectra of FDU‐HOF‐5 before and after the adsorption of DCP (the inset shows the images of FDU‐HOF‐5 before and after the adsorption of DCP taken under a daylight lamp); g) The fluorescence spectra of FDU‐HOF‐5 before and after the adsorption of DCP.

Considering the microporous structure and abundant nitrogen binding sites of FDU‐HOF‐5, it is hypothesized that FDU‐HOF‐5 possesses the capacity to adsorb OPs. Besides DCP (12.23 Å × 6.40 Å × 6.78 Å), we also selected several other OP simulants, including DEP (12.24 Å × 6.67 Å × 5.70 Å), dimethyl phosphite (DMP, 9.73 Å × 5.65 Å × 4.82 Å), trimethyl phosphate (TMP, 9.73 Å × 5.25 Å × 7.86 Å), and dimethyl vinylphosphonate (DVP, 9.60 Å × 5.54 × 7.65 Å) (**Figure** **2**a). We conducted single‐component time‐dependent dynamic vapor sorption (DVS) tests of the above‐mentioned five OP simulants on FDU‐HOF‐5 at 298 K (Figure 2b). The adsorption results demonstrated that FDU‐HOF‐5 possesses the ability to adsorb OP simulants. Due to the size‐sieving effect, the molecular sizes of TMP and DVP are slightly larger than the pore sizes of FDU‐HOF‐5, thus showing poor adsorption behavior. In contrast to the other four OP simulants, FDU‐HOF‐5 demonstrated a higher adsorption capacity for DCP (≈200 mg g^−1^), and the adsorption curve of DCP also manifested a steeper adsorption growth. Notably, the adsorption curve of DCP exhibited a step‐like characteristic, which might be attributed to the interaction between the pores and DCP molecules.^[^ ^44^ ^]^ Meanwhile, we disrupted the pore structure of FDU‐HOF‐5 by grinding (Figures S16 and S17, Supporting Information) and tested the adsorption performance of DCP on the poorly crystallized FDU‐HOF‐5 (Figure S18, Supporting Information). The results showed that its saturated adsorption capacity was only 25% of that of the well‐crystallized FDU‐HOF‐5. This result fully indicates that the excellent adsorption performance of FDU‐HOF‐5 for DCP is closely related to its biomimetic channel structure with high‐density N atom sites.^[^ ^24^ ^]^

Electrostatic potential (ESP) analysis shows that FDU‐HOF‐5,^[^ ^45^, ^46^ ^]^ has a low negative potential area in proximity to the N atom of the triazine ring (Figure 2c). The top of the Cl atom in DCP presents a positive potential (*σ*‐hole, Figure 2d),^[^ ^47^ ^]^ which facilitates electrostatic attraction between DCP and the N atom of the triazine ring in the channel. Utilizing the adsorption functionality of the Materials Studio software suite in conjunction with the Monte–Carlo method,^[^ ^48^ ^]^ the adsorption sites of DCP in the FDU‐HOF‐5 channel were simulated (Scheme 1b; Table S2, Supporting Information). The actual adsorption capacity (≈200 mg g^−1^) is slightly higher than the simulated value (≈180 mg g^−1^), which may be attributed to additional DCP adsorption by mesoporous or microporous structures formed during material accumulation. The OHSs in the channel resemble those of lipase and can effectively recognize DCP, forming multiple interactions with it (Figure S19, Supporting Information). The interaction forces were visualized using the interaction region indicator analysis (Figure S20, Supporting Information).^[^ ^49^ ^]^ Many van der Waals forces exist between DCP and FDU‐HOF‐5, particularly the strong halogen bond and hydrogen bonds formed between Cl and N, which provide a driving force for DCP adsorption. Meanwhile, the analysis of electronic density differences indicates that the formation of halogen bonds and hydrogen bonds reduces the degree of electron sharing in the P─Cl bond, thereby weakening this chemical bond and promoting chlorine dissociation. (Figure 2e).

SEM imaging of the sample adsorbed with DCP revealed no fragmentation or deformation (Figure S21, Supporting Information). PXRD analysis indicated that the crystal structure of FDU‐HOF‐5 was partially damaged after adsorbing DCP. However, the crystal structure of this material could be completely restored by washing with 0.1 m NaOH (Figure S22, Supporting Information). The pore channels of the damaged crystal can also be fully restored at 77 K N_2_ (Figure S23, Supporting Information). Despite this structural resilience, adsorption induced a dramatic color transition from yellow to red, accompanied by a substantial redshift in the UV–vis absorption spectrum (Figure 2f). Concurrent fluorescence measurements revealed a 75 nm redshift with 99.7% quenching efficiency (Figure 2g), thus demonstrating strong host‐guest interactions between FDU‐HOF‐5 and DCP. Intriguingly, the fluorescence color changes were fully reversible following 0.1 m NaOH treatment (Figure S24, Supporting Information), highlighting the dynamic yet stable nature of the system.

### 2.3 Dual‐Modal Fluorescence and Visualization Detection of DCP

**Figure 3:**
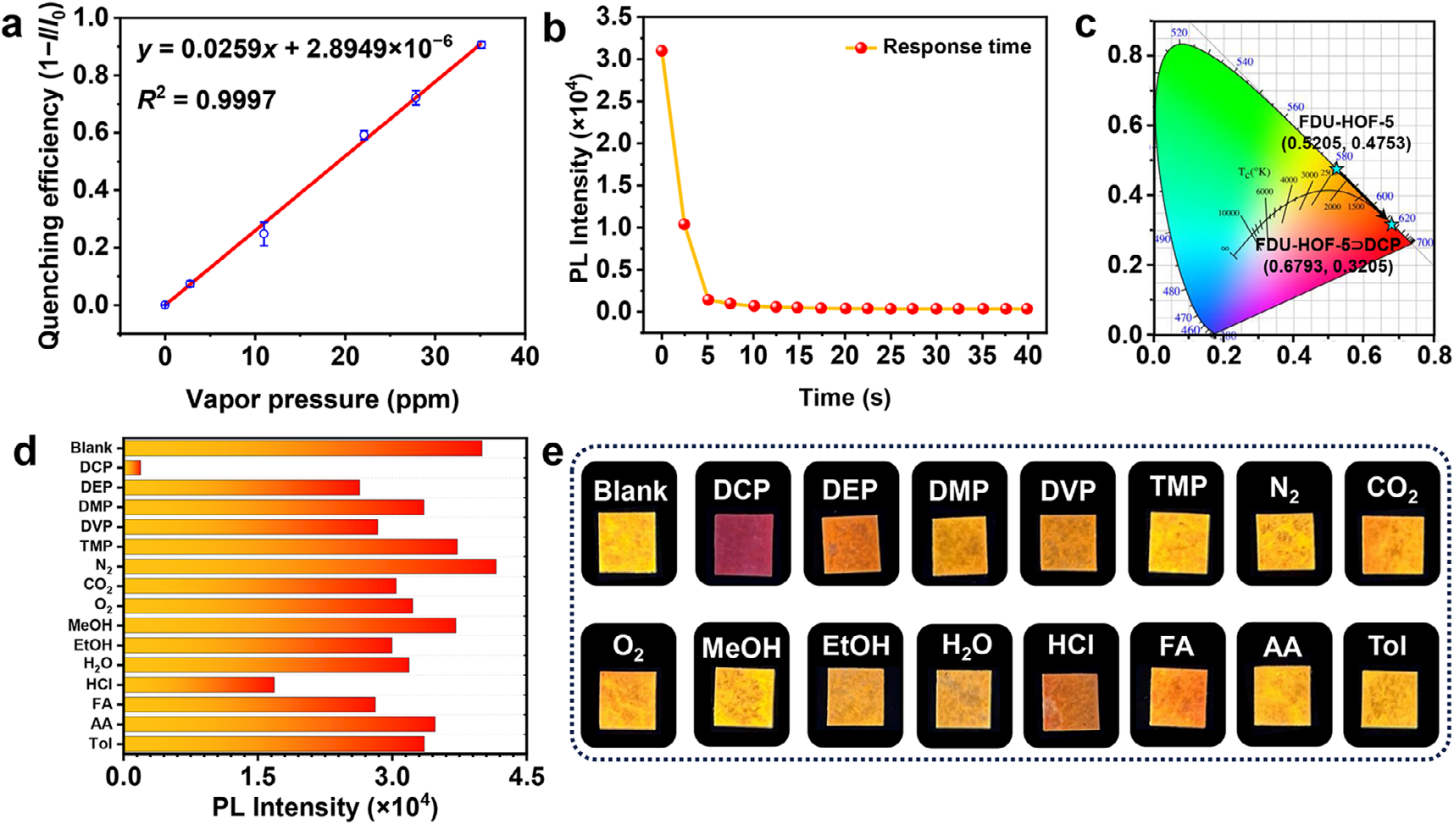
Dual‐modal fluorescence and visualization detection of DCP by FDU‐HOF‐5. a) Linear relationship between quenching efficiency and different concentrations of DCP vapor, error bars represent standard deviation (*n* = 3); b) Response time of FDU‐HOF‐5 to DCP; c) CIE diagram for indicating the emitting color transfer of FDU‐HOF‐5 from (0.5205, 0.4753) to (0.6793, 0.3205) (*λ*_ex_ = 470 nm). d) Histogram of the 580 nm emission peak of FDU‐HOF‐5 after exposure to various gases for 1 min; e) Photographs of FDU‐HOF‐5 after exposure to various gases for 1 min (under UV light, *λ*_ex_ = 365 nm).

As the interaction between DCP and FDU‐HOF‐5 results in significant fluorescence quenching and a distinct visual color change, we believe that FDU‐HOF‐5 has the potential to achieve highly sensitive sensing of DCP. Excited at a wavelength of 470 nm, FDU‐HOF‐5 emits bright yellow light (*λ*_em_ = 580 nm, Figure S25, Supporting Information). Concurrently, exposure to continuous UV light (*λ*_ex_ = 365 nm) for 600 s resulted in a decline in luminescence intensity of ≈10% (Figure S26, Supporting Information). These experiments suggest that FDU‐HOF‐5 could be used as a stable sensing platform. Subsequently, we investigated the fluorescence response of FDU‐HOF‐5 to varying concentrations of DCP. As illustrated in Figure S27 (Supporting Information), the emission intensity of FDU‐HOF‐5 at 580 nm gradually decreased with a noticeable redshift as the DCP concentration increased. A good linear relationship between the luminescence intensity and DCP concentration was observed in the range of 0–35 ppm: *y* = 0.0259*x* + 2.8949×10^−6^, with a correlation coefficient (*R* ^2^) of 0.9997 (**Figure** **3**a). The limit of detection (LOD) was calculated to be 31 ppb based on the 3*σ* criterion (Figure S28, Supporting Information),^[^ ^50^ ^]^ which is below the harmful concentration threshold of DCP.^[^ ^12^ ^]^ The fluorescence signal of FDU‐HOF‐5 stabilized within 5 s after DCP addition (Figure 3b), demonstrating rapid response kinetics. Concurrently, the emission color of the material shifted from yellow to red, with the corresponding Commission Internationale de l'Eclairage (CIE) coordinates changing from (0.5205, 0.4753) to (0.6793, 0.3205) (Figure 3c). The Chromatism (Δ*E*_ab_^*^) was calculated to be 104 (Table S3, Supporting Information),^[^ ^51^ ^]^ indicating a pronounced and readily distinguishable color transition upon DCP exposure. Importantly, this rapid response is not limited to DCP; FDU‐HOF‐5 also exhibits significant fluorescence redshift and quenching within 5 s upon exposure to various other nerve agent simulants (Figure S29, Supporting Information), demonstrating its potential for broad‐spectrum threat detection. This exceptional performance positions FDU‐HOF‐5 favorably against most recently reported MOF and covalent organic framework materials in terms of detection capabilities (Table S4, Supporting Information).

For practical applications, potential interference from environmental gases (N_2_, CO_2_, O_2_, H_2_O) and structural analogs of organophosphorus compounds must be considered. Therefore, we further evaluated the selectivity of FDU‐HOF‐5 toward DCP. Given the outstanding solution processability of HOF materials, portable test strips loaded with FDU‐HOF‐5 were developed (Figure S30, Supporting Information), enabling efficient detection of DCP gas in actual environmental samples. To mimic real‐world conditions, the partial pressures of atmospheric components were maintained within environmentally relevant limits. Formic acid (FA), acetic acid (AA), and dilute HCl were tested at 200 ppm (equivalent to the saturated vapor pressure of typical organophosphorus compounds at 298 K), while other analytes were evaluated at their respective saturated vapor pressures at 298 K. After 1 min of exposure (Figure S31, Supporting Information), only DCP induced the most significant fluorescence quenching and distinct color deepening (Figure 3a,b), confirming that FDU‐HOF‐5 can specifically identify DCP even in complex environments, showcasing excellent selectivity and anti‐interference capability. This remarkable selectivity stems from its 1D channels rich in N atoms, which feature OHSs capable of mimicking the “pockets” where lipases bind to their substrates. Specifically, these hydrogen‐bonding sites can selectively recognize DCP and effectively distinguish it from other structurally similar OPs (Scheme 1b).

### 2.4 Possible Mechanisms of Capture, Sensing, and Degradation

**Figure 4:**
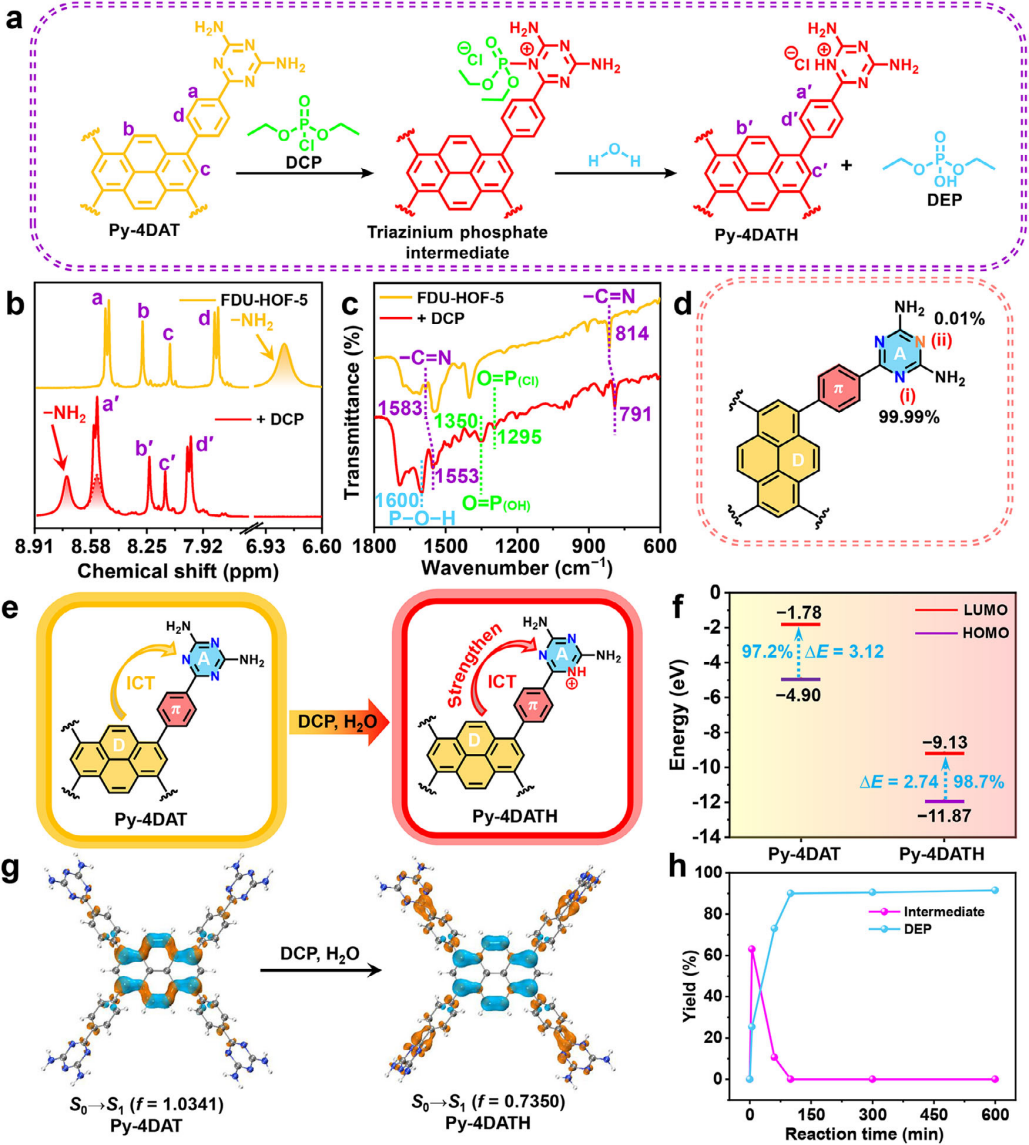
The reaction mechanism of FDU‐HOF‐5 and DCP. a) Possible reaction mechanism of FDU‐HOF‐5 with DCP; b) ^1^H NMR spectra of FDU‐HOF‐5 and after reaction with DCP; c) FT‐IR spectra of FDU‐HOF‐5 before and after adsorption of DCP; d) Two possible protonation sites in Py‐4DAT: i and ii; e) Schematic illustration of the ICT mechanism for the fluorescence variation of FDU‐HOF‐5 induced by DCP; f) The LUMO and HOMO energy levels of Py‐4DAT (left) and Py‐4DATH (right); g) “Electron (brown area)” ‐ “hole (blue area)” analysis of Py‐4DAT (left) and Py‐4DATH (right); h) Images of the generation efficiency of the intermediate and DEP over time.

FDU‐HOF‐5 exhibits excellent adsorption performance and rapid response characteristics toward DCP. Coupled with its significant fluorescence quenching, the evident redshift phenomenon, and reversible color change, this indicates that there is not only a strong host‐guest interaction between FDU‐HOF‐5 and DCP, but also a possible specific chemical recognition. Based on previous reports, we propose a two‐step mechanism (**Figure** **4**a) for the molecular interaction between FDU‐HOF‐5 and DCP:^[^ ^52^, ^53^ ^]^ The N atom in the triazine ring initiates a nucleophilic attack on DCP, forming a reactive intermediate which undergoes hydrolysis, resulting in the protonation of the triazine ring and the conversion of DCP into the non‐toxic DEP. Experimental validation commenced with ^1^H NMR analysis (Figure 4b; Table S5, Supporting Information). Before the reaction, the −NH_2_ group of Py‐4DAT appeared as a single broad peak at *δ* = 6.82 ppm (integration area 15.98), indicating the presence of two chemically equivalent −NH_2_ groups. After exposure to DCP, this −NH_2_ signal shifted downfield and split into two peaks in the *δ* = 8.55 / 8.72 ppm region, with their integration areas reduced by approximately half to ≈8.04. Meanwhile, the corresponding proton signal (1) on the benzene ring also underwent a downfield shift, and its integration area increased from 8.06 to 16.08. This suggests that protonation rendered the two −NH_2_ groups chemically inequivalent, resulting in two signals of equal area, one of which overlaps precisely with the shifted benzene ring proton signal (1′). This trend is entirely consistent with the observations made after treating the sample with HCl, collectively confirming that protonation of the triazine ring is the final step of the reaction. Fourier transform infrared (FT‐IR) spectroscopy further revealed structural changes through redshifted triazine vibrations (1583 → 1553, 814 → 791 cm^−1^; Figure 4c),^[^ ^54^ ^]^ indicative of protonation‐induced distortion. According to the Boltzmann distribution analysis (Table S6, Supporting Information), the conformation corresponding to the protonation site shown in Figure 4d (i) dominates the distribution, accounting for 99.99%.^[^ ^55^ ^]^ Additionally, the electrostatic potential energy analysis reveals that the electrostatic potential energy at this site is lower compared to other possible protonation sites (−31.18 kcal mol^−1^; Figure 2c), further indicating that it is an electron‐rich region, which aligns with reactivity trends.

The protonation of the triazine ring resulted in fluorescence quenching and a redshift of the emission peak, which can be attributed to enhanced ICT, consistent with literature reports (Figure 4e).^[^ ^56^, ^57^ ^]^ Density functional theorycalculations revealed a decrease in the energy gap between the highest occupied molecular orbital (HOMO) and the lowest unoccupied molecular orbital (LUMO) upon protonation (Figure 4f; Figure S33, Supporting Information), leading to a longer absorption wavelength and the observed fluorescence redshift.^[^ ^58^ ^]^ Furthermore, the analysis of hole (blue area) and electron (brown area) distributions showed that in the FDU‐HOF‐5 ligand (Figure 4g), both were primarily localized on the pyrene ring, corresponding to a weak ICT character during the *S*_0_ → *S*_1_ transition.^[^ ^59^ ^]^ After protonation, the hole distribution remained largely unchanged, whereas the electron density shifted along the π‐bridge toward the protonated triazine ring. This resulted in increased spatial separation between holes and electrons, accompanied by a decrease in oscillator strength (*f*) from 1.0341 to 0.7350.^[^ ^60^ ^]^ These results collectively indicate that protonation enhances the ICT character of the ligand. Meanwhile, the strong *π–π* stacking interaction between the layers of FDU‐HOF‐5 leads to a significant contraction of the framework upon the reaction of DCP with the triazine rings in the channels (Figure S22, Supporting Information). This contraction may be related to the strong halogen bonds and hydrogen bonds formed between DCP and the N atoms in the channels, which intensifies the interlayer electron transfer and further quenches the fluorescence of FDU‐HOF‐5.^[^ ^61^ ^]^

The protonation of the triazine ring simultaneously generates the non‐toxic DEP, which also indicates that FDU‐HOF‐5 can degrade DCP through this reaction process. The ^31^P NMR, which used triphenyl phosphate (*δ* = −17.45 ppm) as an internal standard, elucidated the detoxification pathway of DCP (Figure S34, Supporting Information). Within 5 min of mixing DCP with FDU‐HOF‐5, the characteristic peak (*δ* = 2.14 ppm) disappeared, replaced by a transient intermediate peak (*δ* = 7.63 ppm) attributed to triazinium phosphate derivatives.^[^ ^62^ ^]^ The instantaneous generation of this intermediate is consistent with the rapid response behavior of FDU‐HOF‐5 to DCP (Figure 3d). After 100 min, this intermediate vanished, yielding non‐toxic DEP (*δ* = 2.17 ppm) with 91.5% conversion efficiency (Figure 4h). The relatively long hydrolysis process indicates that the diffusion and mass transfer of DCP and water molecules within the pore channels are rather slow. The hygroscopic behavior of FDU‐HOF‐5 under low‐pressure conditions (*p*/*p*_0_ < 0.2; Figure S35, Supporting Information) facilitated the water‐driven environmental detoxification process. FT‐IR confirmed DEP formation via O═P_(OH)_ (1350 cm^−1^) and P─O─H (1600 cm^−1^) absorption bands (Figure 4c).^[^ ^63^, ^64^, ^65^ ^]^ Collectively, spectral analysis, theoretical calculations, and kinetic tracking validate the rationality of the proposed reaction mechanism.

### 2.5 Performance of FDU‐HOF‐5 Loaded on Fabrics

**Figure 5:**
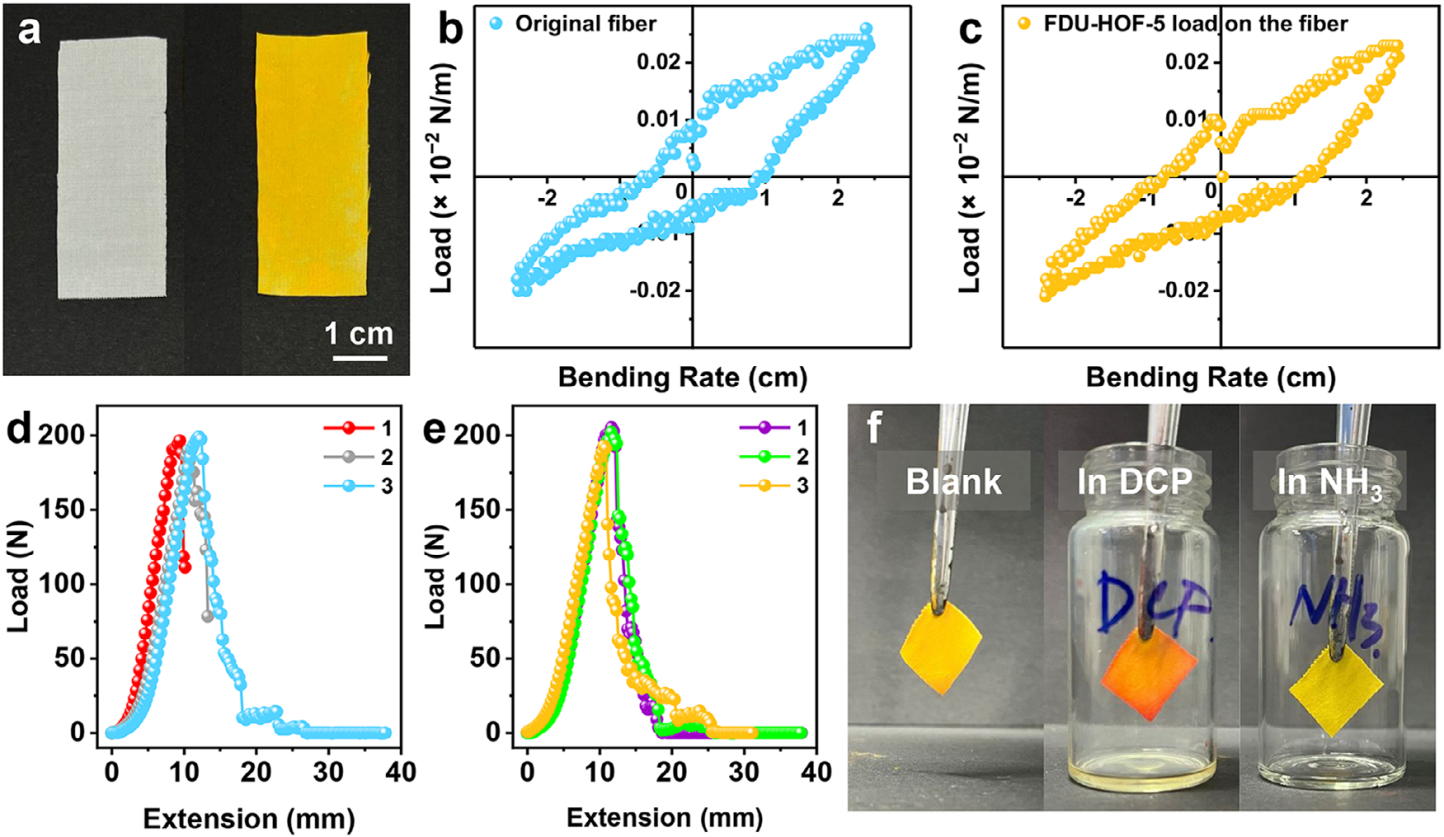
Performance demonstration of FDU‐HOF‐5 loaded on fabrics. a) Photos of the original fabric (left) and FDU‐HOF‐5‐loaded fabric (right); Bending load curve of b) original fabric, and c) FDU‐HOF‐5‐loaded fabric; Tensile strength of d) FDU‐HOF‐5‐loaded fabric, and e) FDU‐HOF‐5‐loaded fabric after bending; f) Photos of the fabric before any treatment (Blank, left), after steam fumigation with DCP (In DCP, middle), and after further fumigation with NH_3_ (In NH_3_, right).

Taking advantage of the excellent solution processability of HOF materials,^[^ ^66^, ^67^ ^]^ FDU‐HOF‐5 was loaded onto fabric with a loading amount of 4.99 wt.% (**Figure** **5**a; Figure S36, Supporting Information). It was demonstrated that the material exhibited similar flexibility before and after being loaded with FDU‐HOF‐5. This is evident from the fact that the maximum loads were ≈0.00025 N m^−1^ at a bending rate of 2.5 cm (Figure 5b,c). SEM images of the original, loaded, and loaded fabric after bending were captured, respectively. After FDU‐HOF‐5 was loaded onto the fiber, the smooth surface was covered with intensive HOF particles (Figure S37a,b, Supporting Information). These particles were found to fix the fabric even after 500 cycles of folding testing (Figure S37c, Supporting Information). As shown in Figure 5d,e, Tables S7 and S8 (Supporting Information), the tensile strengths of the original fabric and FDU‐HOF‐5‐loaded fabric were 200.04 N / 2.5 and 195.25 N / 2.5 cm, respectively. The FDU‐HOF‐5‐loaded fabric possesses a near tensile strain (15.05%), which is similar to that of the original fabric (14.48%). These results indicate that the presence of HOF does not affect the fabric's intrinsic mechanical strength and toughness. The FDU‐HOF‐5‐loaded fabric responded to DCP vapor by changing color from yellow to red within ≈3 s, serving as a visual confirmation of the vapor's presence (Figure 5f, left and middle). Following a treatment in NH_3_ for ≈3 s, the color could almost completely recover (Figure 5f, right). This reversible response could be repeated at least five times (Video S1, Supporting Information). In conclusion, the FDU‐HOF‐5‐loaded fabric demonstrated excellent response performance and outstanding cycling stability to DCP. More importantly, this successful fabrication of a flexible, HOF‐based fabric sensor underscores its practical potential for on‐site, instrument‐free detection via a rapid visual color change.

## 3 Conclusion

Inspired by the recognition mechanism of amino acid residues within the “pocket” of lipase for OP compounds through multiple interactions, this study developed FDU‐HOF‐5, an HOF based on a DAT‐functionalized pyrene building block. The nitrogen‐rich DAT motifs direct the assembly of the framework via hydrogen‐bonding networks, resulting in enzyme‐mimetic 1D channels enriched with open OHSs that facilitate multi‐point weak interactions with OPs. These OHS‐enriched channels enable FDU‐HOF‐5 to effectively adsorb DCP with exceptional selectivity, distinguishing OP analogs even in complex environments with a rapid response (5 s) and an ultra‐low detection limit (31 ppb). Upon adsorption, the interaction induces 99.7% fluorescence quenching accompanied by a 75 nm redshift and a visible yellow‐to‐red color transition, allowing dual‐mode (fluorescence/visual) detection of DCP. Subsequently, the adsorbed DCP is spontaneously degraded into non‐toxic DEP under ambient humidity, achieving a degradation rate of 91.5%. Leveraging the excellent solution processability of HOFs, FDU‐HOF‐5 was successfully loaded onto textile fibers, which exhibited high stability and showed a visually distinguishable color change within 2 s upon contact with DCP. By integrating “adsorption–detection–degradation” functionalities, this work overcomes the bottleneck of balancing selective recognition and multifunctional synergy in conventional porous materials, providing an innovative strategy for designing smart porous materials aimed at chemical threat prevention and control.

## Conflict of Interest

The authors declare no conflict of interest.

## Supporting information



Supporting Information

Supplementary Video 1

## Data Availability

The data that support the findings of this study are available from the corresponding author upon reasonable request.
