# Supplementary material for: Enzyme‐Inspired Hydrogen‐Bonded Organic Frameworks for Synergistic Capture, Detection, and Degradation of Nerve Agent Simulants
Source: Adv Sci (Weinh). 2025 Nov 23;13(8):e19971. doi: 10.1002/advs.202519971 (PMC12884759; doi:10.1002/advs.202519971)
Supplement: Supplementary file 1 — Supporting Information [file ADVS-13-e19971-s002.pdf]

Supporting Information

Enzyme-Inspired Hydrogen-Bonded Organic Frameworks for Synergistic Capture, Detection, and Degradation of Nerve Agent Simulants

Jiabao Liu†, Guanglai Mo†, Xiangyu Gao, Yingjia Deng, Yijin Wang, Qingyu Niu, Yujie Lei, Bin Fei, Joanne Yip, Zhaozhen Zhang, Jie Wu, Yunbo Bi, Kaikai Ma,* Zhiqiang Li*, and Peng Li**

† J.L. and G.M. contributed equally to this work.

Contents

1. Reagents and instruments. ········ 1

1.1. Materials. ········ 1

1.2. Characterizations. ········ 1

2. Synthesis of Py-4DAT. ········ 2

3. Synthesis of microcrystalline FDU-HOF-5 powder. ········ 2

4. N2 sorption measurements. ········ 2

5. Powder X-ray diffraction (PXRD) analyses. ········ 2

6. FDU-HOF-5 crystal stability test. ········ 2

7. Thermogravimetric analyses (TGA). ········ 3

8. Measurement of Fourier transform infrared (FT-IR) spectra. ········ 3

9. The DVS adsorption curves of FDU-HOF-5 for DCP, DEP, DMP, TMP, and DVP at 298 K. ········ 3

10. The ^{31}P NMR test for the reaction of DCP with FDU-HOF-5 to generate DEP. ········ 3

11. Water sorption measurements. ········ 4

12. Standard procedure of FDU-HOF-5-loaded fabric. ········ 4

13. Folding test of FDU-HOF-5-loaded fabric. ········ 4

14. Measurement of tensile strength. ········ 4

15. Calculation. ········ 4

16. The calculation method of chromatism (ΔE_{ab}^*). ········ 5

17. Tables. ········ 6

Table S1. Fractional atomic coordinates for FDU-HOF-5. ········ 6

Table S2. Fractional atomic coordinates for FDU-HOF-5⊃DCP. ········ 8

Table S3. The Lab values (L_1^*, a_1^*, b_1^* and L_2^*, a_2^*, b_2^*) of ratiometric fluorescent probes. ········ 10

Table S4. Comparison of various fluorescent frameworks for nerve agent simulant. ········ 11

Table S5. The ^{1}H NMR integration changes of FDU-HOF-5 before and after the reaction with DCP. 11

Table S6. Boltzmann populations for different protonation sites. ········ 12

Table S7. Tensile strength of FDU-HOF-5-loaded fabric. ········ 12

Table S8. Tensile strength of FDU-HOF-5-loaded fabric after folding. ········ 12

18. Figures. ········ 13

Figure S1. The D-π-A structure of Py-4DAT. ········ 13

Figure S2. Synthetic route of Py-4DAT. ········ 13

Figure S3. ^{1}H NMR spectra of Py-4DAT (400 MHz, DMSO-d_6). ········ 13

Figure S4. ^{13}C NMR spectra of Py-4DAT (101 MHz, DMSO-d_6). ········ 14

Figure S5. HRMS spectrum of Py-4DAT. ········ 14

Figure S6. The experimental PXRD of FDU-HOF-5. ········ 15

Figure S7. The IFFT image corresponding to the {010} lattice plane of FDU-HOF-5. ················ 15
Figure S8. The lengths of the interplanar spacings of 10 groups of (010) crystal planes. ··············· 16
Figure S9. The theoretical interplanar spacing of the {010} crystal plane. ································ 16
Figure S10. The *sql* topology diagram of FDU-HOF-5. ··· 17
Figure S11. The Connolly surface of FDU-HOF-5. ·· 17
Figure S12. Pore size distribution plots for FDU-HOF-5. ·· 18
Figure S13. PXRD patterns of FDU-HOF-5 after 24 h treatment at different temperatures. ··········· 18
Figure S14. TGA curves of FDU-HOF-5. ··· 19
Figure S15. PXRD patterns of FDU-HOF-5 after treatment with different solvents for 2 days. ······· 19
Figure S16. The PXRD pattern of the poorly crystalline FDU-HOF-5. ································· 20
Figure S17. N_2 adsorption-desorption isotherms of the poorly crystalline FDU-HOF-5 at 77 K. ······· 20
Figure S18. The DCP adsorption curve of the poorly crystalline FDU-HOF-5. ························· 21
Figure S19. The multiple interactions formed between FDU-HOF-5 and DCP . ························ 21
Figure S20. IRI analysis for FDU-HOF-5⊃DCP. ·· 22
Figure S21. The SEM images of FDU-HOF-5 before and after the adsorption of DCP. ················ 22
Figure S22. The PXRD spectra of FDU-HOF-5 after the adsorption of DCP and after treatment with 0.1 M NaOH. ·· 23
Figure S23. The 77 K N_2 adsorption isotherm of the repaired crystal. ··································· 23
Figure S24. Photographs of FDU-HOF-5 (left), FDU-HOF-5⊃DCP (middle) and FDU-HOF-5⊃DCP treated with 0.1 M NaOH (right), (a) under daylight, (b) under UV (λ_{ex} = 365 nm) light.24
Figure S25. The fluorescence excitation and emission spectra of FDU-HOF-5. ························ 24
Figure S26. The time-dependent variation of the luminescence intensity of FDU-HOF-5. ············· 25
Figure S27. Fluorescence emission spectra of FDU-HOF-5 under different concentrations of DCP. · 25
Figure S28. Three repeated fluorescent measurements of pristine FDU-HOF-5 solid. ·················· 26
Figure S29. Fluorescence spectra of three organophosphorus simulants (DMCP, DICP, DCNP) before and after interaction with FDU-HOF-5. ·· 26
Figure S30. Flowchart of the preparation of the test strips for detecting DCP. ························· 27
Figure S31. Schematic illustration of the fumigation device. ··· 27
Figure S32. The ^{1}H NMR spectra of Py-4DAT after treatment with DCP and HCl, respectively. ····· 28
Figure S33. The HOMO and the LUMO of Py-4DAT and Py-4DATH. ································· 28
Figure S34. The ^{31}P NMR of the reaction of DCP with FDU-HOF-5 as a function of time. ··········· 29
Figure S35. The water adsorption isotherm of FDU-HOF-5. ·· 29
Figure S36. Standard procedure of FDU-HOF-5-loaded fabric. ··· 30
19. References. ··· 31

1. Reagents and instruments.

1.1. Materials.

All chemicals and solvents used for synthesis were purchased from commercial sources and used as received without any further purification.

1.2. Characterizations.

^{1}H, ^{13}C, and ^{31}P NMR spectra were collected by Bruker AVANCE III 400 MHz spectrometers (Bruker Corporation, Karlsruhe, Germany). High-resolution mass spectrometry (HRMS) was determined by ThermoFisher Q-Exactive Focus (Thermo Fisher Scientific, Waltham, MA, USA). The ^{13}C Solid-state NMR (ssNMR) experiments were performed on a Bruker WB Avance II 400 MHz spectrometer (Bruker Corporation, Karlsruhe, Germany). Powder X-ray diffraction (PXRD) patterns were measured using a Rigaku Smatlab 9 kW diffractometer equipped with a Cu rotating anode X-ray source (Rigaku Corporation, Tokyo, Japan). Fourier transform infrared (FT-IR) spectra were collected by Thermo Fisher Nicolet iS10 (Thermo Fisher Scientific, Waltham, MA, USA). Photoluminescence spectra were obtained on FLS1000 (Edinburgh Instruments, Livingston, United Kingdom). Solid UV-vis spectra were tested with a Lambda 650S UV-vis spectrophotometer (PerkinElmer, Waltham, MA, USA). The N_2 isotherm was measured using BSD-PM2 (Beishide Instrument Technology Co., Beijing, China). Thermogravimetric analyses (TGA) were performed on TGA8000 (PerkinElmer, Waltham, MA, USA). Scanning electron microscope (SEM) images were obtained on a LaB6 VEGA 3 XMU scanning electron microscope (TESCAN, Brno, South Moravia, Czech Republic) at an acceleration voltage of 0.2 – 30 kV. High-resolution cryo-transmission electron microscopy (cryo-TEM) was acquired on JEM-2100F (JEOL, Japan) transmission electron microscope with an accelerating voltage of 200 kV. The dynamic vapor sorption (DVS) test was performed by the BSD Vacuum & Dynamic Vapor/Gas Sorption Analyzer (Beishide Instrument Technology Co., Beijing, China). Water adsorption and desorption isotherms were measured on a JW-ZQ100 (JWGB Sci & Tech Co. Ltd.). The folding test was performed with a pure bending tester (Kato Tech Co., Ltd., Osaka, Japan). Tensile strength measurements were performed with an Instron 4411 (Instron Tech Co., Ltd., High Wycombe, United Kingdom).

2. Synthesis of Py-4DAT.

Synthetic routes for Py-4DAT are shown in Figure S2. Py-4DAT was synthesised via a modified literature method.[1,2] In a 250 mL round-bottom flask, 1,3,6,8-tetrakis(4-cyanophenyl)pyrene (1 g, 1.65 mmol), KOH (90%, 0.5 g), and dicyandiamide (2.77 g, 33.0 mmol) were dissolved in 2-methoxyethanol (50 mL). The mixture was stirred at 140 °C for 48 h under N_2. After cooling to room temperature, the product was precipitated with MeOH, filtered, and washed with boiling water and MeOH. Recrystallization from dimethyl sulfoxide (DMSO)/acetone (v/v = 1:40) yielded orange crystals (1.2 g, 77%). ^{1}H NMR (400 MHz, DMSO-d_6) δ 8.48 (d, J = 7.9 Hz, 8H), 8.28 (s, 4H), 8.11 (s, 2H), 7.84 (d, J = 8.0 Hz, 8H), 6.82 (s, 16H). ^{13}C NMR (101 MHz, DMSO-d_6) δ 169.95, 167.53, 142.61, 136.70, 136.46, 130.42, 129.47, 128.03, 127.55, 125.42 (m). HRMS $[M+H]^+$ calcd. for $C_{52}H_{39}N_{20}^+$ 943.3661; found: 943.3660.

3. Synthesis of microcrystalline FDU-HOF-5 powder.

Microcrystalline FDU-HOF-5 powder was synthesized by dissolving Py-4DAT (1 g, 1.06 mmol) in DMSO (30 mL) and rapidly pouring acetone (500 mL) into the solution. The mixture was kept static for 24 h, yielding yellow rod-shaped crystals (900 – 930 mg, 90 – 93%).

4. N_2 sorption measurements.

100 mg of FDU-HOF-5 were activated at 100 °C under a vacuum for 12 h before adsorption measurements. The N_2 isotherm measurements were performed at 77 K.

5. Powder X-ray diffraction (PXRD) analyses.

PXRD patterns were measured at 40 kV, 200 mA for Cu Kα radiation (λ = 1.5406 Å), with a scan speed of 0.2 s/step and a step size of 0.05°.

6. FDU-HOF-5 crystal stability test.

15 mg FDU-HOF-5 was soaked in MeOH, EtOH, acetone, ethyl acetate (EA), tetrahydrofuran (THF), 1,4-dioxane, and 10 M NaOH for 2 days. After the supernatant was removed, the samples were collected and dried in a vacuum oven before the PXRD test. 15 mg of FDU-HOF-5 was placed under vacuum at 60, 100, 150, and 200 °C, respectively, for 24 h, followed by PXRD testing.

7. Thermogravimetric analyses (TGA).

TGA was performed from 25 – 800°C with a heating rate of 10 °C/min in N_2.

8. Measurement of Fourier transform infrared (FT-IR) spectra.

Using a KBr pellet method, the FT-IR spectra of powder samples were recorded in the 400 – 4000 cm^{-1} frequency region.

9. The DVS adsorption curves of FDU-HOF-5 for DCP, DEP, DMP, TMP, and DVP at 298 K.

Before the test, a 30 mg sample of FDU-HOF-5 was placed in a crucible and activated by nitrogen purging at 373 K until a constant weight was achieved. Once cooled to 298 K, the DVS test was initiated. The sample was then separately purged with DCP (260.53 ppm), DEP (180.26 ppm), DMP (1332.35 ppm), TMP (1118.18 ppm), and DVP (5039.47 ppm) (balanced with N_2) at a flow rate of 40 sccm until its weight stabilized.

10. The ^{31}P NMR test for the reaction of DCP with FDU-HOF-5 to generate DEP.

6 mg of triphenyl phosphate (TPP, an internal standard substance with $\delta = -17.45$) was dissolved in 600 μL of CD_3OD to prepare a 10 mg/mL TPP CD_3OD solution for standby. The aforementioned solution was transferred to a 2 mL transparent small glass vial. Subsequently, 36 mg of FDU-HOF-5 was dispersed in the solution. After 10 min of sonication, 5.45 μL of DCP was added, and the mixture was shaken uniformly until a uniform color (from yellow to red) was achieved. Then, 1 μL of deionized water was added and shaken evenly. Subsequently, all the evenly shaken dispersion was transferred to a nuclear magnetic resonance tube, and phosphorus spectrum tests were conducted successively at 5, 60, 100, 300, and 600 min, respectively. Likewise, without adding FDU-HOF-5 and keeping other methods unchanged, the ^{31}P NMR of DCP is tested.

11. Water sorption measurements.

Water adsorption and desorption isotherms were measured on a JW-ZQ100 (JWGB Sci & Tech Co. Ltd.) at 298 K. Vapor sources were degassed prior to the measurements. Activation was performed by heating the FDU-HOF-5 at 90°C for 12 h under dynamic vacuum on a Smart VacPrep. The temperature was maintained at 298 K using an Iso Controller.

12. Standard procedure of FDU-HOF-5-loaded fabric.

0.0360 g of Py-4DAT was dissolved in a surface dish containing 0.8 mL of DMSO. The solution was evenly dropped onto a white fabric with a mass of 0.3590 g, and the surface dish was placed in a beaker containing 10 mL of acetone. The beaker was sealed and left to stand for 4 days; then, the fabric was washed with acetone and dried by air. Yellow fabric loaded with HOF (FDU-HOF-5-loaded fabric) was obtained and weighed 0.3769 g (Amount of loading: 4.99%).

13. Folding test of FDU-HOF-5-loaded fabric.

The FDU-HOF-5-loaded fabric was anchored on the bending tester and rotated 500 cycles (time of a cycle: 10 s).

14. Measurement of tensile strength.

Three standard fabric stripes (size of fabric: 2.5 cm × 13 cm) of the original fabric and the FDU-HOF-5-loaded fabric were cut, respectively. The tensile strengths of the above fabric were measured according to the ASTM D5035 Standard.

15. Calculation.

All calculations have been carried out with Gaussian 16 (Rev. C.01).[3] The geometry was fully optimized using density functional theory (DFT) methods, at the B3LYP-D3(BJ) level with the standard 6-311^{++}G(d, p) basis set. At the same level, excited states were calculated via TDDFT. Multiwfn and VMD software performed orbital energy level analysis,

hole-electron analysis, ESP evaluation algorithm, IRI analysis, and electron density difference map.[4-8] The structure model of FDU-HOF-5 was constructed through theoretical simulation using the Materials Studio software package.[9] Based on the simulated unit cell parameters, the Pawley refinement of the PXRD pattern was conducted.[10] The adsorption simulation of DCP in FDU-HOF-5 was conducted using the Materials Studio software using the Monte Carlo method.[9] In the study, the COMPASSII force field was adopted, and periodic boundary conditions were applied. The electrostatic interactions within the FDU-HOF-5 material were evaluated using the Ewald summation method. Meanwhile, the van der Waals interactions between DCP and FDU-HOF-5 were calculated using the Lennard-Jones potential function, with a cutoff distance set at 18.5 Å. The atomic charge parameters of DCP molecules and FDU-HOF-5 were obtained through the COMPASSII force field in the Forcite module.

16. The calculation method of chromatism (ΔE_{ab}^*).

Luminescent images of reported sensors in the absence and presence of DCP vapor were analyzed using Adobe Photoshop software. The Lab values (L_1^*, a_1^*, b_1^*, L_2^*, a_2^*, and b_2^*) were obtained using the eyedropper tool and presented in Table S3. ΔE_{ab}^*, representing the quantitative difference between two colors, can be defined as:[11]

$$\Delta E_{ab}^* = [(L_1^* - L_2^*)^2 + (a_1^* - a_2^*)^2 + (b_1^* - b_2^*)^2]^{1/2}$$

17. Tables.

Table S1. Fractional atomic coordinates for FDU-HOF-5.

Number	Label	Charge	Sybyl Type	X	Y	Z	Symm. op.
1	N108	0	N.3	0.2797	-0.07895	-1.03954	x,y,z
2	H109	0	H	0.19372	-0.13576	-1.04656	x,y,z
3	H110	0	H	0.42533	-0.07324	-0.99521	x,y,z
4	C1	0	C.2	0.76752	0.42576	-1.03508	x,y,z
5	C2	0	C.2	1.04594	0.54659	-1.08734	x,y,z
6	C3	0	C.2	0.93954	0.5955	-1.20608	x,y,z
7	C4	0	C.2	0.92325	0.53658	-1.14893	x,y,z
8	C5	0	C.2	0.76934	0.46582	-1.15161	x,y,z
9	H6	0	H	0.69613	0.45517	-1.1992	x,y,z
10	C7	0	C.2	0.70186	0.40807	-1.09669	x,y,z
11	C8	0	C.2	1.2673	0.60916	-1.0816	x,y,z
12	H9	0	H	1.36775	0.65003	-1.12434	x,y,z
13	C10	0	C.2	0.95398	0.49316	-1.03024	x,y,z
14	N11	0	N.2	0.90838	0.75045	-1.43254	x,y,z
15	C12	0	C.2	0.96439	0.67407	-1.20328	x,y,z
16	H13	0	H	0.97735	0.69022	-1.15634	x,y,z
17	N14	0	N.2	0.98533	0.84192	-1.36732	x,y,z
18	C15	0	C.2	1.35746	0.62246	-1.0226	x,y,z
19	H16	0	H	1.52478	0.67305	-1.022	x,y,z
20	C17	0	C.2	0.94003	0.71296	-1.31989	x,y,z
21	C18	0	C.2	0.58125	0.33496	-1.10426	x,y,z
22	C19	0	C.2	0.96938	0.73059	-1.2578	x,y,z
23	H20	0	H	0.99216	0.78955	-1.25278	x,y,z
24	N21	0	N.2	0.95358	0.88092	-1.48108	x,y,z
25	C22	0	C.2	0.91025	0.57943	-1.2694	x,y,z
26	H23	0	H	0.88877	0.52052	-1.27468	x,y,z
27	C24	0	C.2	0.90686	0.63571	-1.32417	x,y,z
28	H25	0	H	0.88382	0.62035	-1.37137	x,y,z
29	N26	0	N.3	1.03706	0.97734	-1.41689	x,y,z
30	C27	0	C.2	0.94467	0.77023	-1.37506	x,y,z
31	N28	0	N.3	0.88937	0.78999	-1.55089	x,y,z
32	C29	0	C.2	0.98993	0.89609	-1.42202	x,y,z
33	C30	0	C.2	0.91286	0.80804	-1.48484	x,y,z
34	C31	0	C.2	0.65771	0.26724	-1.05729	x,y,z
35	H32	0	H	0.80988	0.27079	-1.01371	x,y,z
36	C33	0	C.2	0.39313	0.32497	-1.16113	x,y,z
37	H34	0	H	0.32871	0.37451	-1.19895	x,y,z
38	C35	0	C.2	0.28973	0.25493	-1.17006	x,y,z
39	H36	0	H	0.14595	0.25049	-1.21433	x,y,z
40	C37	0	C.2	0.54781	0.1972	-1.06511	x,y,z
41	H38	0	H	0.60965	0.14747	-1.02736	x,y,z
42	C39	0	C.2	0.36036	0.18905	-1.1218	x,y,z
43	N40	0	N.3	-0.23085	0.03505	-1.24989	x,y,z
44	N41	0	N.2	0.02947	-0.01843	-1.14428	x,y,z
45	C42	0	C.2	-0.0264	0.04381	-1.1905	x,y,z
46	N43	0	N.2	0.07771	0.11382	-1.18484	x,y,z
47	C44	0	C.2	0.24716	0.11819	-1.12953	x,y,z

Number	Label	Charge	Sybyl Type	X	Y	Z	Symm. op.
48	N45	0	N.2	0.31425	0.05847	-1.0811	x,y,z
49	C46	0	C.2	0.1987	-0.00941	-1.09032	x,y,z
50	H94	0	H	1.06663	0.99128	-1.36873	x,y,z
51	H95	0	H	1.04215	1.02314	-1.46126	x,y,z
52	H96	0	H	0.86219	0.73034	-1.55548	x,y,z
53	H97	0	H	0.8994	0.83637	-1.59489	x,y,z
54	H98	0	H	-0.32567	-0.02191	-1.25523	x,y,z
55	H99	0	H	-0.28649	0.08555	-1.28818	x,y,z
56	N108	0	N.3	1.7203	1.07895	-0.96046	2-x,1-y,-2-z
57	H109	0	H	1.80628	1.13576	-0.95344	2-x,1-y,-2-z
58	H110	0	H	1.57467	1.07324	-1.00479	2-x,1-y,-2-z
59	C1	0	C.2	1.23248	0.57424	-0.96492	2-x,1-y,-2-z
60	C2	0	C.2	0.95406	0.45341	-0.91266	2-x,1-y,-2-z
61	C3	0	C.2	1.06046	0.4045	-0.79392	2-x,1-y,-2-z
62	C4	0	C.2	1.07675	0.46342	-0.85107	2-x,1-y,-2-z
63	C5	0	C.2	1.23066	0.53418	-0.84839	2-x,1-y,-2-z
64	H6	0	H	1.30387	0.54483	-0.8008	2-x,1-y,-2-z
65	C7	0	C.2	1.29814	0.59193	-0.90331	2-x,1-y,-2-z
66	C8	0	C.2	0.7327	0.39084	-0.9184	2-x,1-y,-2-z
67	H9	0	H	0.63225	0.34997	-0.87566	2-x,1-y,-2-z
68	C10	0	C.2	1.04602	0.50684	-0.96976	2-x,1-y,-2-z
69	N11	0	N.2	1.09162	0.24955	-0.56746	2-x,1-y,-2-z
70	C12	0	C.2	1.03561	0.32593	-0.79672	2-x,1-y,-2-z
71	H13	0	H	1.02265	0.30978	-0.84366	2-x,1-y,-2-z
72	N14	0	N.2	1.01467	0.15808	-0.63268	2-x,1-y,-2-z
73	C15	0	C.2	0.64254	0.37754	-0.9774	2-x,1-y,-2-z
74	H16	0	H	0.47522	0.32695	-0.978	2-x,1-y,-2-z
75	C17	0	C.2	1.05997	0.28704	-0.68011	2-x,1-y,-2-z
76	C18	0	C.2	1.41875	0.66504	-0.89574	2-x,1-y,-2-z
77	C19	0	C.2	1.03062	0.26941	-0.7422	2-x,1-y,-2-z
78	H20	0	H	1.00784	0.21045	-0.74722	2-x,1-y,-2-z
79	N21	0	N.2	1.04642	0.11908	-0.51892	2-x,1-y,-2-z
80	C22	0	C.2	1.08975	0.42057	-0.7306	2-x,1-y,-2-z
81	H23	0	H	1.11123	0.47948	-0.72532	2-x,1-y,-2-z
82	C24	0	C.2	1.09314	0.36429	-0.67583	2-x,1-y,-2-z
83	H25	0	H	1.11618	0.37965	-0.62863	2-x,1-y,-2-z
84	N26	0	N.3	0.96294	0.02266	-0.58311	2-x,1-y,-2-z
85	C27	0	C.2	1.05533	0.22977	-0.62494	2-x,1-y,-2-z
86	N28	0	N.3	1.11063	0.21001	-0.44911	2-x,1-y,-2-z
87	C29	0	C.2	1.01007	0.10391	-0.57798	2-x,1-y,-2-z
88	C30	0	C.2	1.08714	0.19196	-0.51516	2-x,1-y,-2-z
89	C31	0	C.2	1.34229	0.73276	-0.94271	2-x,1-y,-2-z
90	H32	0	H	1.19012	0.72921	-0.98629	2-x,1-y,-2-z
91	C33	0	C.2	1.60687	0.67503	-0.83887	2-x,1-y,-2-z
92	H34	0	H	1.67129	0.62549	-0.80105	2-x,1-y,-2-z
93	C35	0	C.2	1.71027	0.74507	-0.82994	2-x,1-y,-2-z
94	H36	0	H	1.85405	0.74951	-0.78567	2-x,1-y,-2-z
95	C37	0	C.2	1.45219	0.8028	-0.93489	2-x,1-y,-2-z
96	H38	0	H	1.39035	0.85253	-0.97264	2-x,1-y,-2-z
97	C39	0	C.2	1.63964	0.81095	-0.8782	2-x,1-y,-2-z
98	N40	0	N.3	2.23085	0.96495	-0.75011	2-x,1-y,-2-z
99	N41	0	N.2	1.97053	1.01843	-0.85572	2-x,1-y,-2-z

Number	Label	Charge	Sybyl Type	X	Y	Z	Symm. op.
100	C42	0	C.2	2.0264	0.95619	-0.8095	2-x,1-y,-2-z
101	N43	0	N.2	1.92229	0.88618	-0.81516	2-x,1-y,-2-z
102	C44	0	C.2	1.75284	0.88181	-0.87047	2-x,1-y,-2-z
103	N45	0	N.2	1.68575	0.94153	-0.9189	2-x,1-y,-2-z
104	C46	0	C.2	1.8013	1.00941	-0.90968	2-x,1-y,-2-z
105	H94	0	H	0.93337	0.00872	-0.63127	2-x,1-y,-2-z
106	H95	0	H	0.95785	-0.02314	-0.53874	2-x,1-y,-2-z
107	H96	0	H	1.13781	0.26966	-0.44452	2-x,1-y,-2-z
108	H97	0	H	1.1006	0.16363	-0.40511	2-x,1-y,-2-z
109	H98	0	H	2.32567	1.02191	-0.74477	2-x,1-y,-2-z
110	H99	0	H	2.28649	0.91445	-0.71182	2-x,1-y,-2-z

Table S2. Fractional atomic coordinates for FDU-HOF-5⊃DCP.

Number	Label	Charge	Sybyl Type	X	Y	Z	Symm. op.
1	N1	0	N.3	-0.7203	-0.07895	-0.03954	x,y,z
2	H2	0	H	-0.80628	-0.13576	-0.04656	x,y,z
3	H3	0	H	-0.57467	-0.07324	0.00479	x,y,z
4	C4	0	C.2	-0.23248	0.42576	-0.03508	x,y,z
5	C5	0	C.2	0.04594	0.54659	-0.08734	x,y,z
6	C6	0	C.2	-0.06046	0.5955	-0.20608	x,y,z
7	C7	0	C.2	-0.07675	0.53658	-0.14893	x,y,z
8	C8	0	C.2	-0.23066	0.46582	-0.15161	x,y,z
9	H9	0	H	-0.30387	0.45517	-0.1992	x,y,z
10	C10	0	C.2	-0.29814	0.40807	-0.09669	x,y,z
11	C11	0	C.2	0.2673	0.60916	-0.0816	x,y,z
12	H12	0	H	0.36775	0.65003	-0.12434	x,y,z
13	C13	0	C.2	-0.04602	0.49316	-0.03024	x,y,z
14	N14	0	N.2	-0.09162	0.75045	-0.43254	x,y,z
15	C15	0	C.2	-0.03561	0.67407	-0.20328	x,y,z
16	H16	0	H	-0.02265	0.69022	-0.15634	x,y,z
17	N17	0	N.2	-0.01467	0.84192	-0.36732	x,y,z
18	C18	0	C.2	0.35746	0.62246	-0.0226	x,y,z
19	H19	0	H	0.52478	0.67305	-0.022	x,y,z
20	C20	0	C.2	-0.05997	0.71296	-0.31989	x,y,z
21	C21	0	C.2	-0.41875	0.33496	-0.10426	x,y,z
22	C22	0	C.2	-0.03062	0.73059	-0.2578	x,y,z
23	H23	0	H	-0.00784	0.78955	-0.25278	x,y,z
24	N24	0	N.2	-0.04642	0.88092	-0.48108	x,y,z
25	C25	0	C.2	-0.08975	0.57943	-0.2694	x,y,z
26	H26	0	H	-0.11123	0.52052	-0.27468	x,y,z
27	C27	0	C.2	-0.09314	0.63571	-0.32417	x,y,z
28	H28	0	H	-0.11618	0.62035	-0.37137	x,y,z
29	N29	0	N.3	0.03706	0.97734	-0.41689	x,y,z
30	C30	0	C.2	-0.05533	0.77023	-0.37506	x,y,z
31	N31	0	N.3	-0.11063	0.78999	-0.55089	x,y,z
32	C32	0	C.2	-0.01007	0.89609	-0.42202	x,y,z
33	C33	0	C.2	-0.08714	0.80804	-0.48484	x,y,z
34	C34	0	C.2	-0.34229	0.26724	-0.05729	x,y,z
35	H35	0	H	-0.19012	0.27079	-0.01371	x,y,z

Number	Label	Charge	Sybyl Type	X	Y	Z	Symm. op.
36	C36	0	C.2	-0.60687	0.32497	-0.16113	x,y,z
37	H37	0	H	-0.67129	0.37451	-0.19895	x,y,z
38	C38	0	C.2	-0.71027	0.25493	-0.17006	x,y,z
39	H39	0	H	-0.85405	0.25049	-0.21433	x,y,z
40	C40	0	C.2	-0.45219	0.1972	-0.06511	x,y,z
41	H41	0	H	-0.39035	0.14747	-0.02736	x,y,z
42	C42	0	C.2	-0.63964	0.18905	-0.1218	x,y,z
43	N43	0	N.3	-1.23085	0.03505	-0.24989	x,y,z
44	N44	0	N.2	-0.97053	-0.01843	-0.14428	x,y,z
45	C45	0	C.2	-1.0264	0.04381	-0.1905	x,y,z
46	N46	0	N.2	-0.92229	0.11382	-0.18484	x,y,z
47	C47	0	C.2	-0.75284	0.11819	-0.12953	x,y,z
48	N48	0	N.2	-0.68575	0.05847	-0.0811	x,y,z
49	C49	0	C.2	-0.8013	-0.00941	-0.09032	x,y,z
50	H50	0	H	0.06663	0.99128	-0.36873	x,y,z
51	H51	0	H	0.04215	1.02314	-0.46126	x,y,z
52	H52	0	H	-0.13781	0.73034	-0.55548	x,y,z
53	H53	0	H	-0.1006	0.83637	-0.59489	x,y,z
54	H54	0	H	-1.32567	-0.02191	-0.25523	x,y,z
55	H55	0	H	-1.28649	0.08555	-0.28818	x,y,z
56	N56	0	N.3	0.7203	1.07895	0.03954	x,y,z
57	H57	0	H	0.80628	1.13576	0.04656	x,y,z
58	H58	0	H	0.57467	1.07324	-0.00479	x,y,z
59	C59	0	C.2	0.23248	0.57424	0.03508	x,y,z
60	C60	0	C.2	-0.04594	0.45341	0.08734	x,y,z
61	C61	0	C.2	0.06046	0.4045	0.20608	x,y,z
62	C62	0	C.2	0.07675	0.46342	0.14893	x,y,z
63	C63	0	C.2	0.23066	0.53418	0.15161	x,y,z
64	H64	0	H	0.30387	0.54483	0.1992	x,y,z
65	C65	0	C.2	0.29814	0.59193	0.09669	x,y,z
66	C66	0	C.2	-0.2673	0.39084	0.0816	x,y,z
67	H67	0	H	-0.36775	0.34997	0.12434	x,y,z
68	C68	0	C.2	0.04602	0.50684	0.03024	x,y,z
69	N69	0	N.2	0.09162	0.24955	0.43254	x,y,z
70	C70	0	C.2	0.03561	0.32593	0.20328	x,y,z
71	H71	0	H	0.02265	0.30978	0.15634	x,y,z
72	N72	0	N.2	0.01467	0.15808	0.36732	x,y,z
73	C73	0	C.2	-0.35746	0.37754	0.0226	x,y,z
74	H74	0	H	-0.52478	0.32695	0.022	x,y,z
75	C75	0	C.2	0.05997	0.28704	0.31989	x,y,z
76	C76	0	C.2	0.41875	0.66504	0.10426	x,y,z
77	C77	0	C.2	0.03062	0.26941	0.2578	x,y,z
78	H78	0	H	0.00784	0.21045	0.25278	x,y,z
79	N79	0	N.2	0.04642	0.11908	0.48108	x,y,z
80	C80	0	C.2	0.08975	0.42057	0.2694	x,y,z
81	H81	0	H	0.11123	0.47948	0.27468	x,y,z
82	C82	0	C.2	0.09314	0.36429	0.32417	x,y,z
83	H83	0	H	0.11618	0.37965	0.37137	x,y,z
84	N84	0	N.3	-0.03706	0.02266	0.41689	x,y,z
85	C85	0	C.2	0.05533	0.22977	0.37506	x,y,z
86	N86	0	N.3	0.11063	0.21001	0.55089	x,y,z
87	C87	0	C.2	0.01007	0.10391	0.42202	x,y,z

Number	Label	Charge	Sybyl Type	X	Y	Z	Symm. op.
88	C88	0	C.2	0.08714	0.19196	0.48484	x,y,z
89	C89	0	C.2	0.34229	0.73276	0.05729	x,y,z
90	H90	0	H	0.19012	0.72921	0.01371	x,y,z
91	C91	0	C.2	0.60687	0.67503	0.16113	x,y,z
92	H92	0	H	0.67129	0.62549	0.19895	x,y,z
93	C93	0	C.2	0.71027	0.74507	0.17006	x,y,z
94	H94	0	H	0.85405	0.74951	0.21433	x,y,z
95	C95	0	C.2	0.45219	0.8028	0.06511	x,y,z
96	H96	0	H	0.39035	0.85253	0.02736	x,y,z
97	C97	0	C.2	0.63964	0.81095	0.1218	x,y,z
98	N98	0	N.3	1.23085	0.96495	0.24989	x,y,z
99	N99	0	N.2	0.97053	1.01843	0.14428	x,y,z
100	C100	0	C.2	1.0264	0.95619	0.1905	x,y,z
101	N101	0	N.2	0.92229	0.88618	0.18484	x,y,z
102	C102	0	C.2	0.75284	0.88181	0.12953	x,y,z
103	N103	0	N.2	0.68575	0.94153	0.0811	x,y,z
104	C104	0	C.2	0.8013	1.00941	0.09032	x,y,z
105	H105	0	H	-0.06663	0.00872	0.36873	x,y,z
106	H106	0	H	-0.04215	-0.02314	0.46126	x,y,z
107	H107	0	H	0.13781	0.26966	0.55548	x,y,z
108	H108	0	H	0.1006	0.16363	0.59489	x,y,z
109	H109	0	H	1.32567	1.02191	0.25523	x,y,z
110	H110	0	H	1.28649	0.91445	0.28818	x,y,z
111	P111	0	P.3	0.23514	0.52645	0.48283	x,y,z
112	O112	0	O.2	-0.02306	0.48932	0.43801	x,y,z
113	Cl113	0	Cl	0.01391	0.61907	0.51894	x,y,z
114	O114	0	O.3	0.37199	0.47416	0.55513	x,y,z
115	O115	0	O.3	0.63227	0.56134	0.44806	x,y,z
116	C116	0	C.3	0.60791	0.41038	0.55518	x,y,z
117	H117	0	H	0.85129	0.43404	0.5302	x,y,z
118	H118	0	H	0.47423	0.3713	0.52926	x,y,z
119	C119	0	C.3	0.65543	0.59018	0.37656	x,y,z
120	H120	0	H	0.93403	0.58524	0.36711	x,y,z
121	H121	0	H	0.49611	0.55214	0.35316	x,y,z
122	C122	0	C.3	0.66771	0.37423	0.62576	x,y,z
123	H123	0	H	0.84096	0.32686	0.62961	x,y,z
124	H124	0	H	0.79193	0.41509	0.65087	x,y,z
125	H125	0	H	0.41813	0.35289	0.64965	x,y,z
126	C126	0	C.3	0.54392	0.67127	0.35662	x,y,z
127	H127	0	H	0.58959	0.69255	0.30469	x,y,z
128	H128	0	H	0.26344	0.67555	0.36581	x,y,z
129	H129	0	H	0.69616	0.70754	0.38277	x,y,z

Table S3. The Lab values (L_1^*, a_1^*, b_1^* and L_2^*, a_2^*, b_2^*) of ratiometric fluorescent probes.

	L_1^*	a_1^*	b_1^*	L_2^*	a_2^*	b_2^*
FDU-HOF-5	98	−16	92	42	64	57

Table S4. Comparison of various fluorescent frameworks for nerve agent simulant.

Type	Name	Test substances	LOD	Response time	Ref.
MOF	SNNU-357	DCP	0.5 ppb	—	[12]
MOF	UiO-66-OPD@CNF	DCNP	0.685 ppb	1 s	[13]
MOF	Ag-AuNCs@UiO-66-NH_2	DCP	1.02 ppb	—	[14]
MOF	Me_4BOPHY-1@ZIF-8	DCP	1.13 ppb	3 s	[15]
MOF	EuMOF@CNF	DCP	2.8 ppb	1 s	[16]
MOF	Cu^{2+}@UiO-66-NH_2/OPD	DCP	6.65 ppb	—	[17]
HOF	FDU-HOF-5	DCP	37 ppb	5 s	This Work
COF	TAPB-PDA-COFs	DCP	40 ppb	1.1 s	[18]
MOF	SNNU-267	DCP	0.04 ppm	10 s	[19]
MOF	SNNU-266	DCP	0.08 ppm	10 s	[19]
MOF	Cd-ANTBI	DCP	0.52 ppm	—	[20]
MOF	Eu-PyIPA	DCP	0.7 ppm	20 s	[21]
MOF	UiO-66–NH_2@Aga	DCP	1.16 ppm	—	[22]
MOF	Eu-BTC@UiO-66-NH_2	DCP	24.76 ppm	—	[23]
MOF	Hf-DUT-52	DCP	9 nM	5 s	[24]
MOF	Eu-CTTB-MOF	DCP	20.97 nM	1 s	[25]
COF	Tb@ETTADFP-COOH	DCNP	0.0249 μM	10 s	[26]
COF	DAFB-DCTP	DCNP	13.5 μM	—	[27]
MOF	Tb^{3+}@UiO-66-DPA	DCP	68 μM	7 min	[28]

Table S5. The ^{1}H NMR integration changes of FDU-HOF-5 before and after the reaction with DCP.

	1	2	3	4	$-NH_2$
FDU-HOF-5	8.06	3.95	2.00	8.00	15.98
+DCP	16.08	4.06	2.00	8.09	8.04

Table S6. Calculated free energy differences and corresponding Boltzmann populations for different protonation sites, Figure 4d(i) and Figure 4d(ii).

Index	ΔG (kcal/mol)	Q_i (Relat)	Percent
Figure 4d(i)	0	1	99.99%
Figure 4d(ii)	5.860	5.062×10^{-5}	0.01%

Table S7. Tensile strength of FDU-HOF-5-loaded fabric.

	Maximum load (N)	Tensile extension at maximum load (mm)	Tensile strain at maximum load (%)
1	205.23	11.67	15.56
2	202.14	11.60	15.47
3	192.75	10.60	14.13
**Mean**	**200.04**	**11.29**	**15.05**

Table S8. Tensile strength of FDU-HOF-5-loaded fabric after folding.

	Maximum load (N)	Tensile extension at maximum load (mm)	Tensile strain at maximum load (%)
1	196.37	9.41	12.55
2	190.33	11.06	14.75
3	199.06	12.10	16.13
**Mean**	**195.25**	**10.86**	**14.48**

18. Figures.

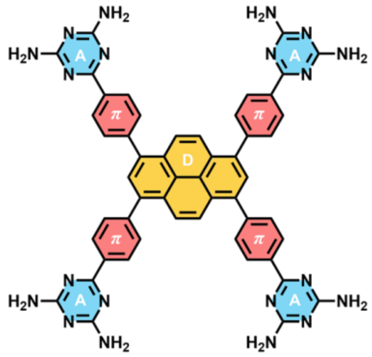

Figure S1. The D-π-A structure of Py-4DAT.

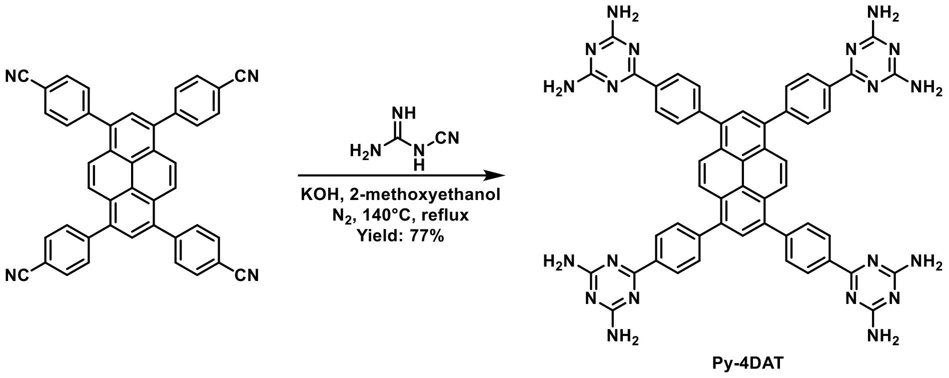

Figure S2. Synthetic route of Py-4DAT.

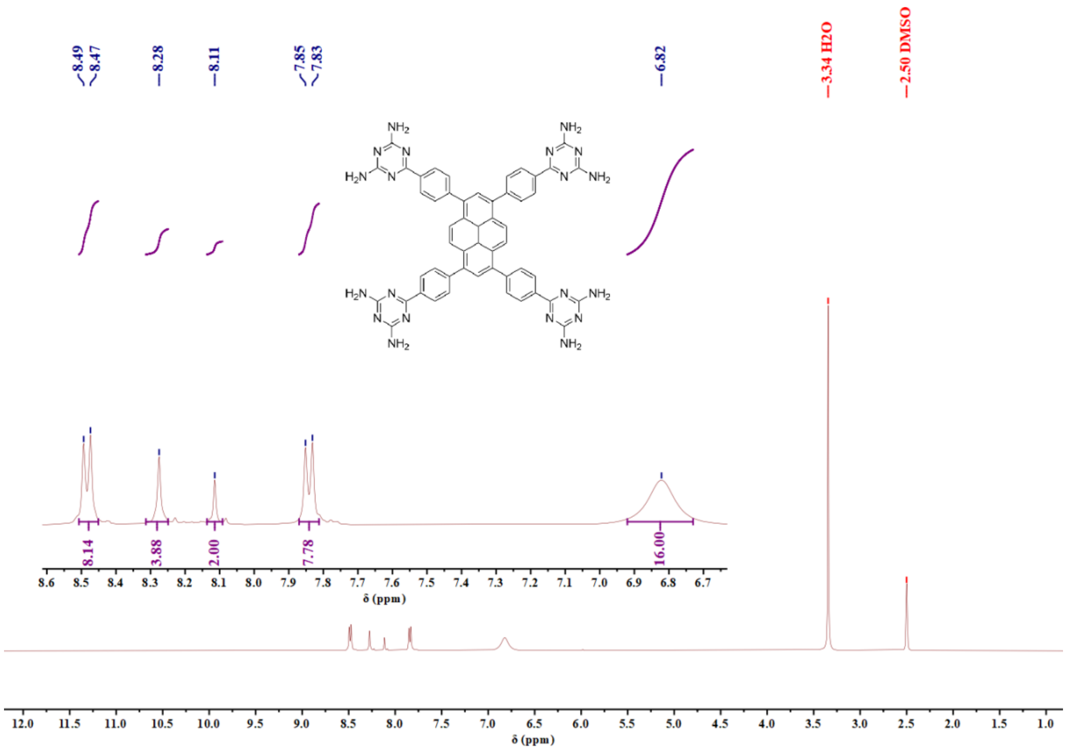

Figure S3. ^{1}H NMR spectra of Py-4DAT (400 MHz, DMSO-d_6).

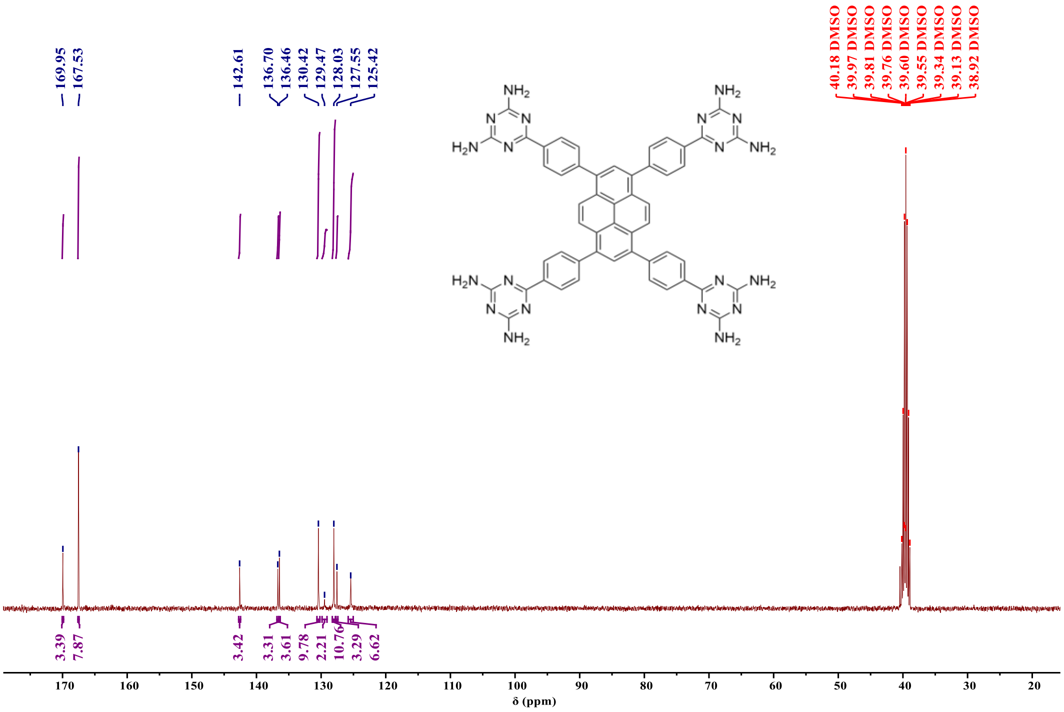

Figure S4. ^{13}C NMR spectra of Py-4DAT (101 MHz, DMSO-d_6).

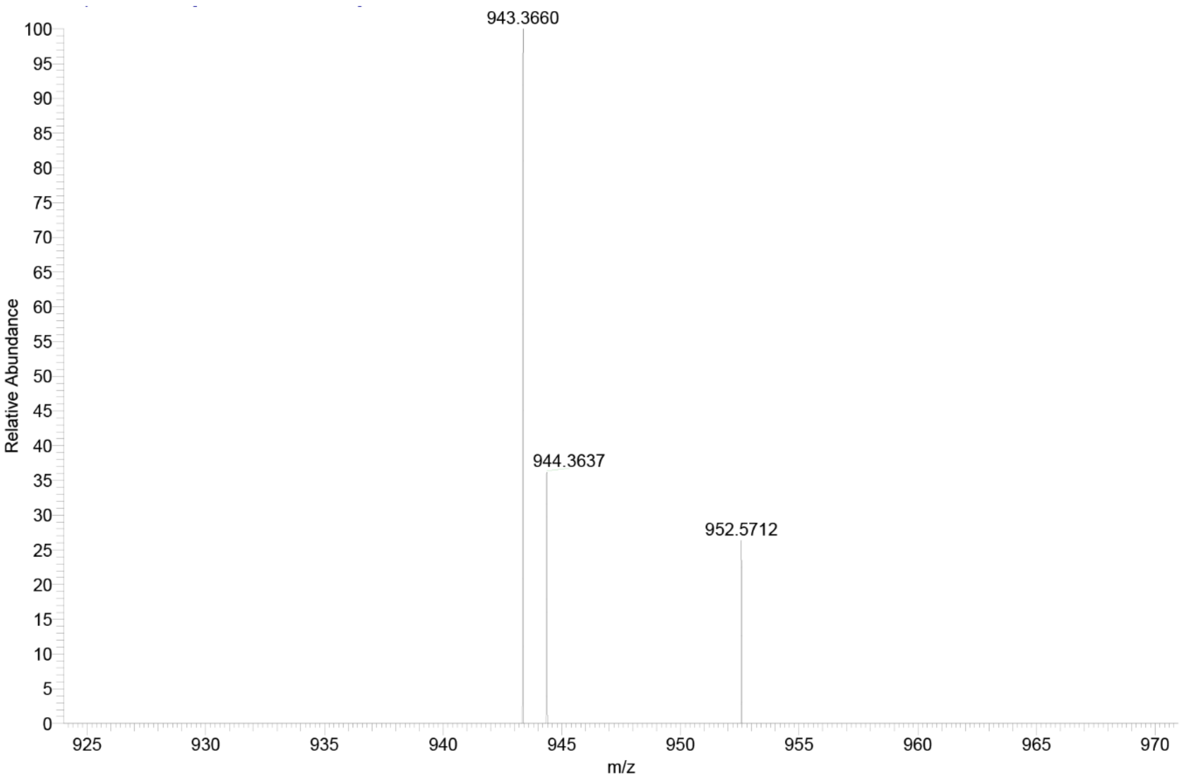

Figure S5. HRMS spectrum of Py-4DAT.

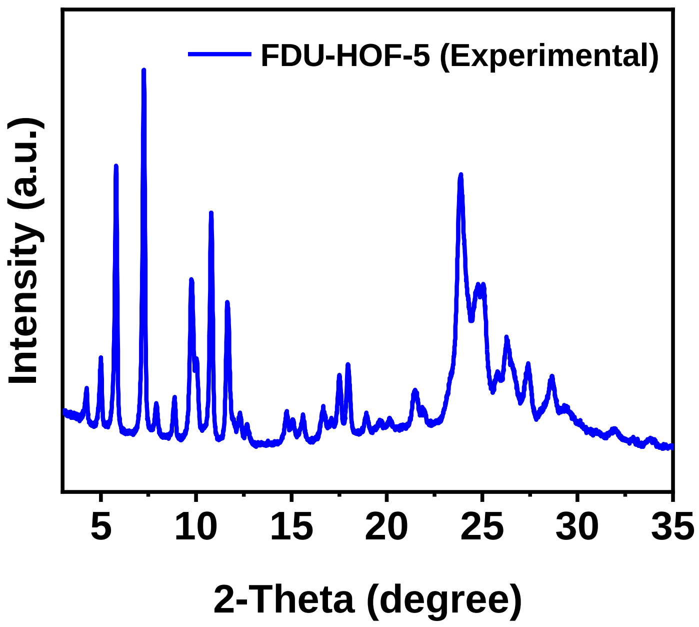

Figure S6. The experimental PXRD of FDU-HOF-5.

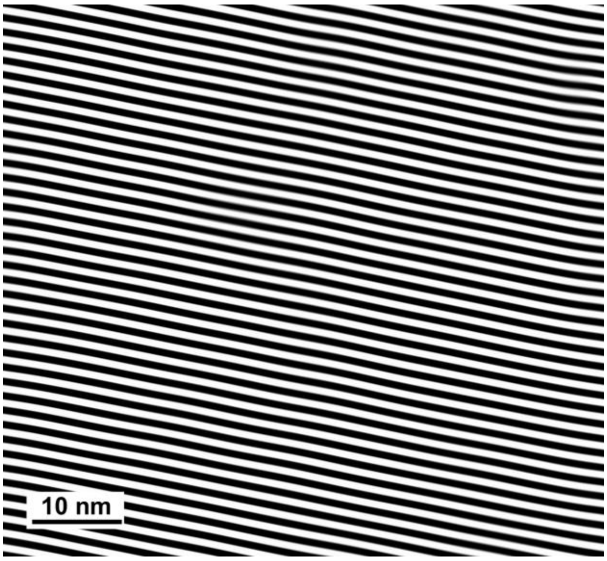

Figure S7. The inverse fast Fourier transform (IFFT) image corresponding to the {010} lattice plane of FDU-HOF-5.

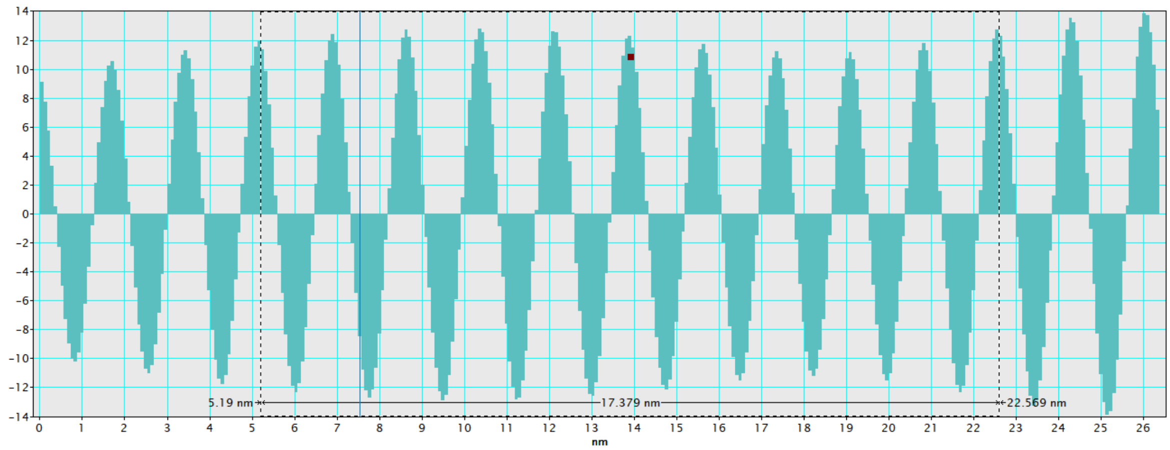

Figure S8. The illustration shows the lengths of the interplanar spacings of 10 groups of (010) crystal planes.

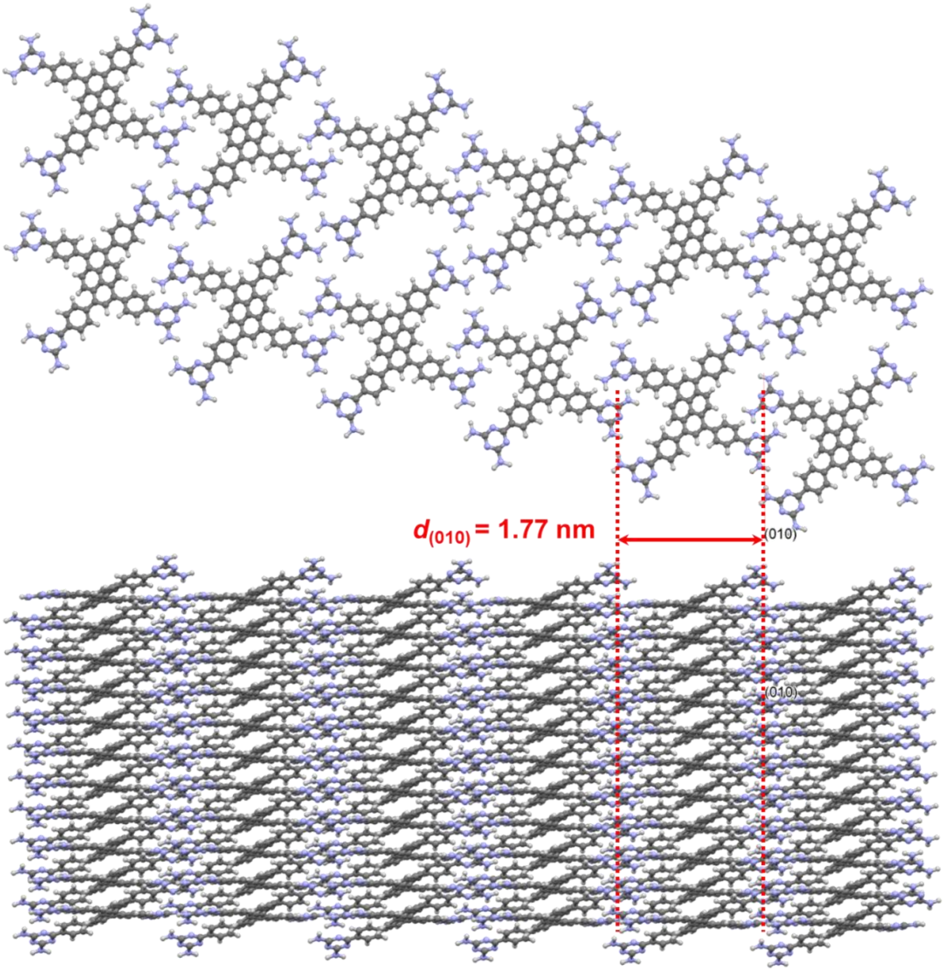

Figure S9. The theoretical interplanar spacing of the {010} crystal plane in the FDU-HOF-5 structure.

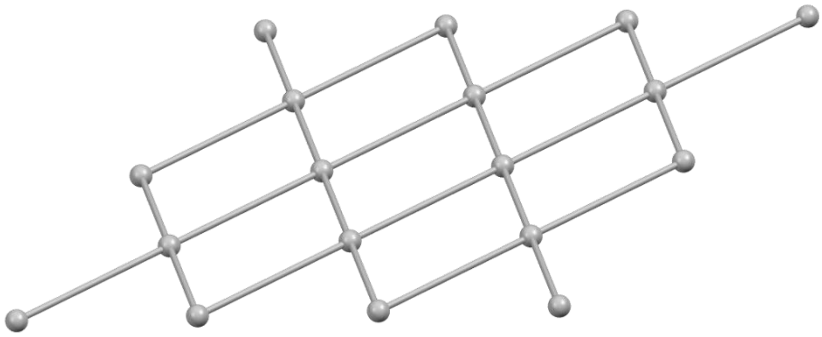

Figure S10. The ***sql*** topology diagram of FDU-HOF-5.

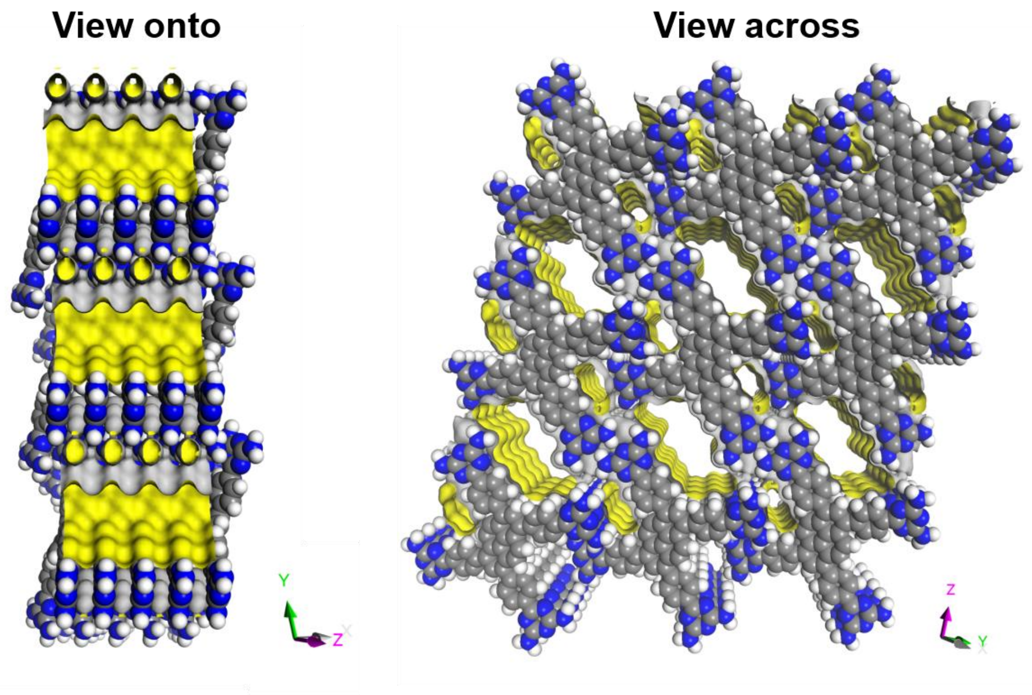

Figure S11. The Connolly surface of FDU-HOF-5 is shown by Materials Studio under view onto (left) and view across (right).

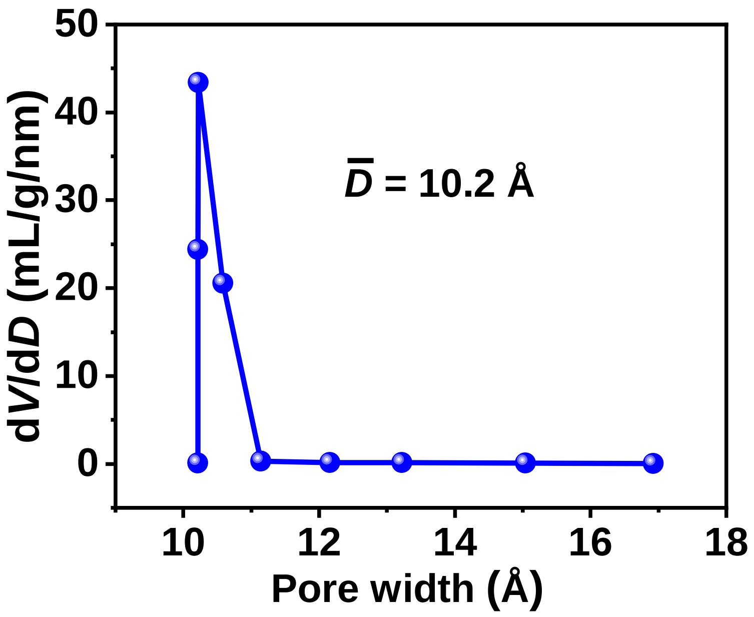

Figure S12. Pore size distribution plots for FDU-HOF-5.

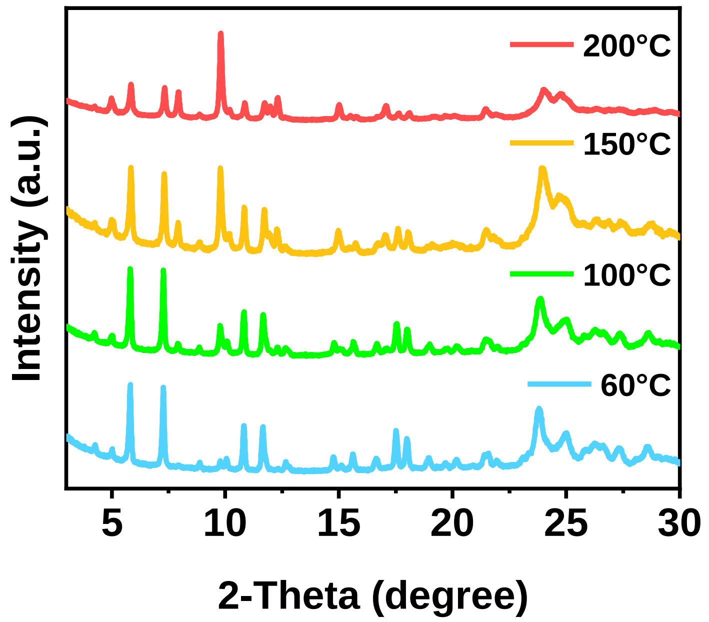

Figure S13. PXRD patterns of FDU-HOF-5 after 24 h treatment at different temperatures.

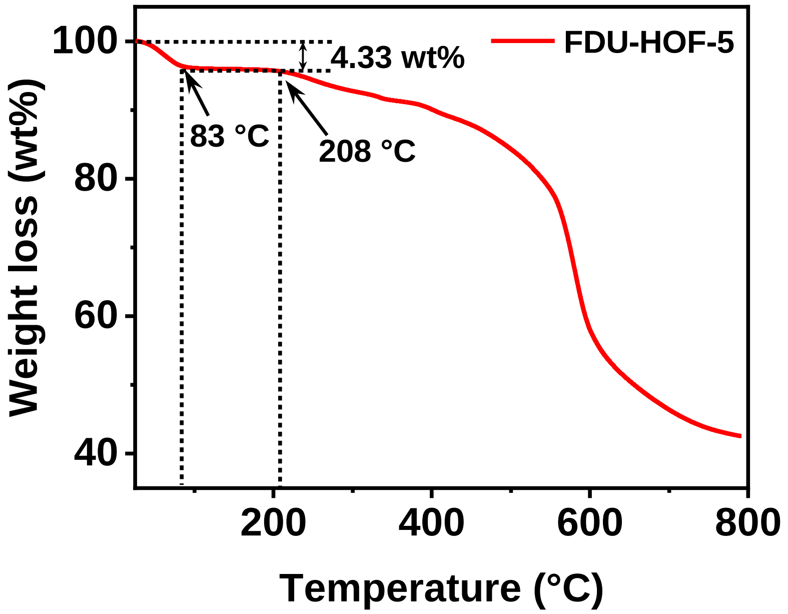

Figure S14. TGA curves of FDU-HOF-5.

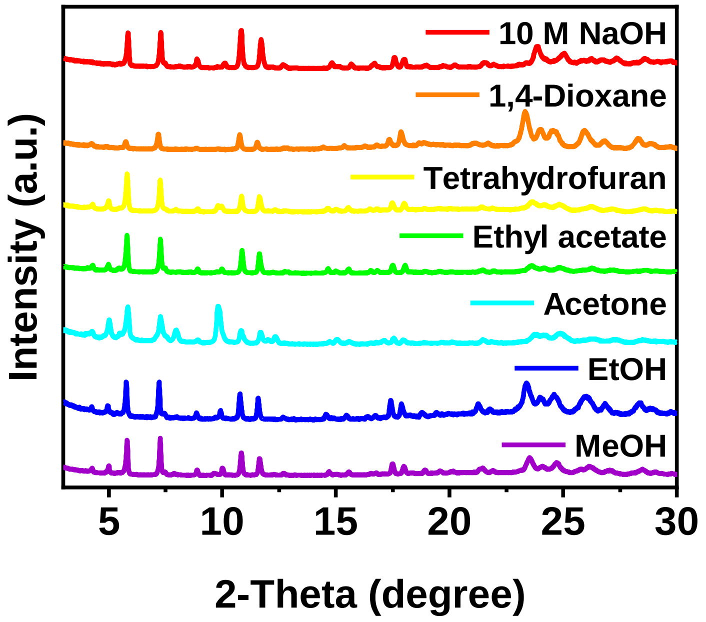

Figure S15. PXRD patterns of FDU-HOF-5 after treatment with different solvents for 2 days.

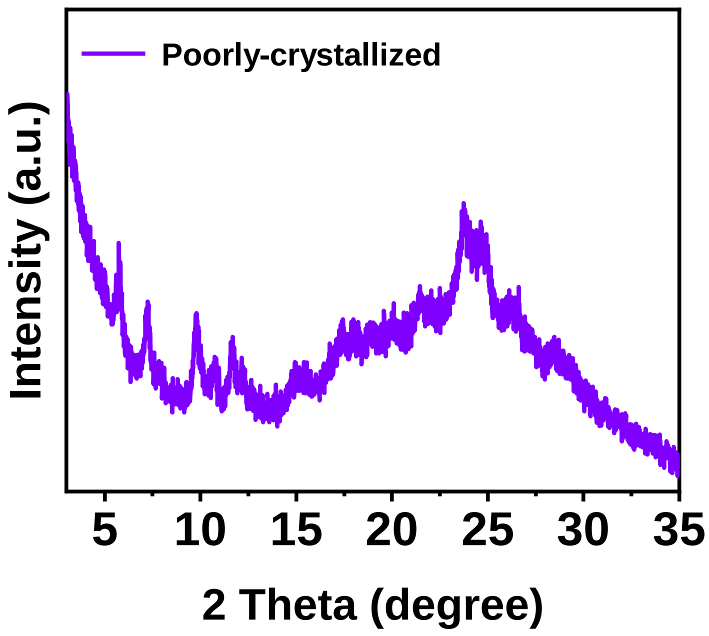

Figure S16. The PXRD pattern of the poorly crystalline FDU-HOF-5.

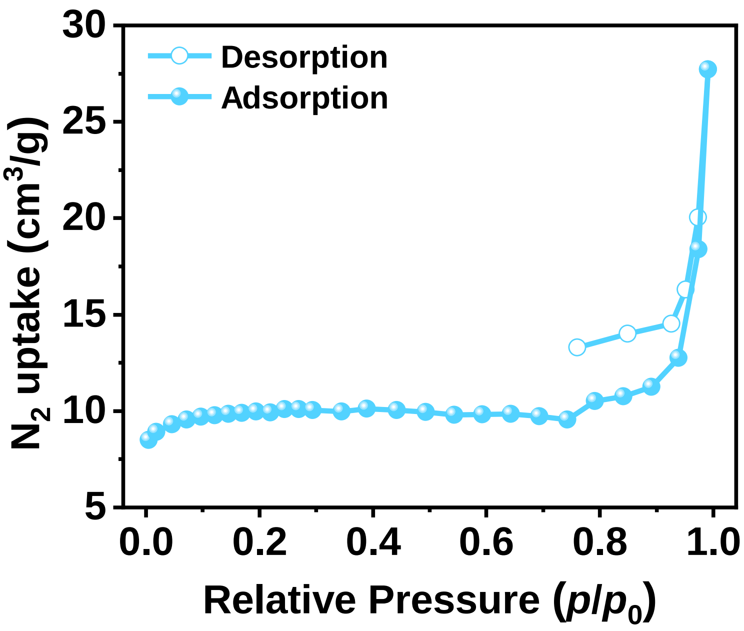

Figure S17. N_2 adsorption-desorption isotherms of the poorly crystalline FDU-HOF-5 at 77 K.

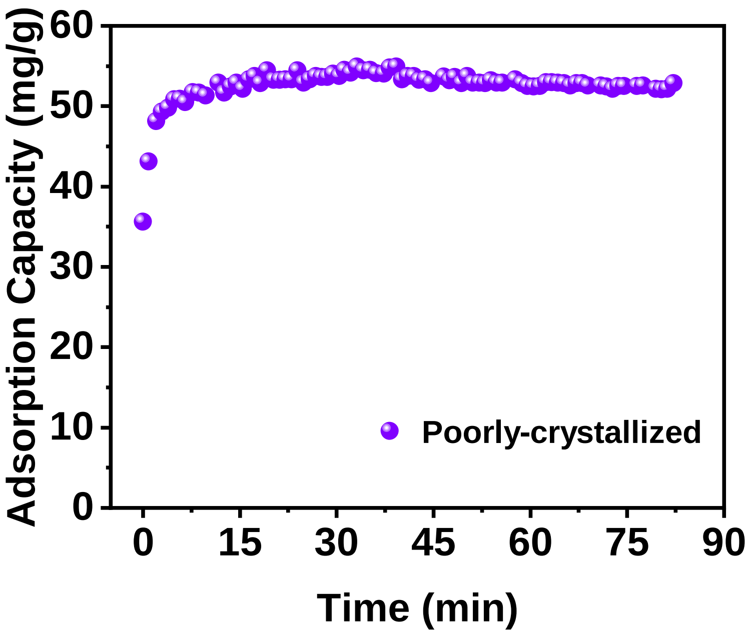

Figure S18. The DCP adsorption curve of the poorly crystalline FDU-HOF-5.

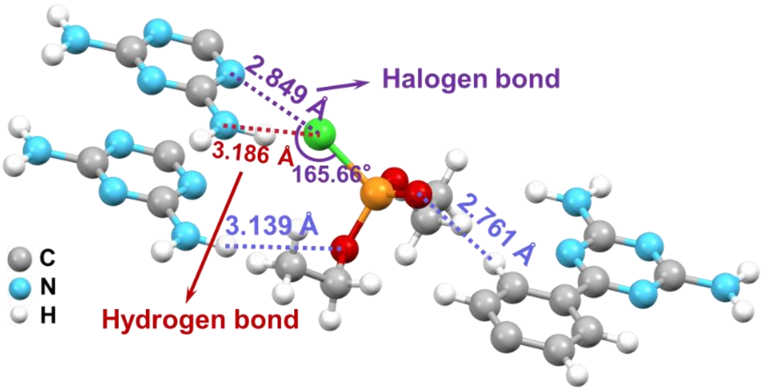

Figure S19. The multiple interactions formed between FDU-HOF-5 and DCP (purple indicates halogen bonds, red indicates hydrogen bonds, and blue indicates Van der Waals interactions).

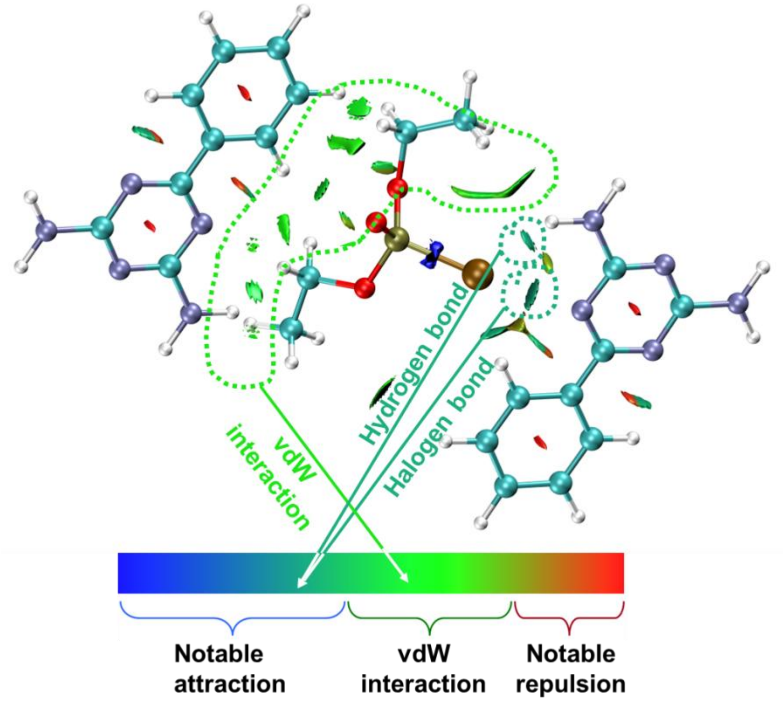

Figure S20. IRI analysis for FDU-HOF-5⊃DCP. Red regions highlight areas of significant repulsion, such as steric effects within rings and cages. Green regions indicate areas dominated by Van der Waals interactions, while blue regions signify notable attractive interactions, including hydrogen bonds, halogen bonds, and chemical bonding.

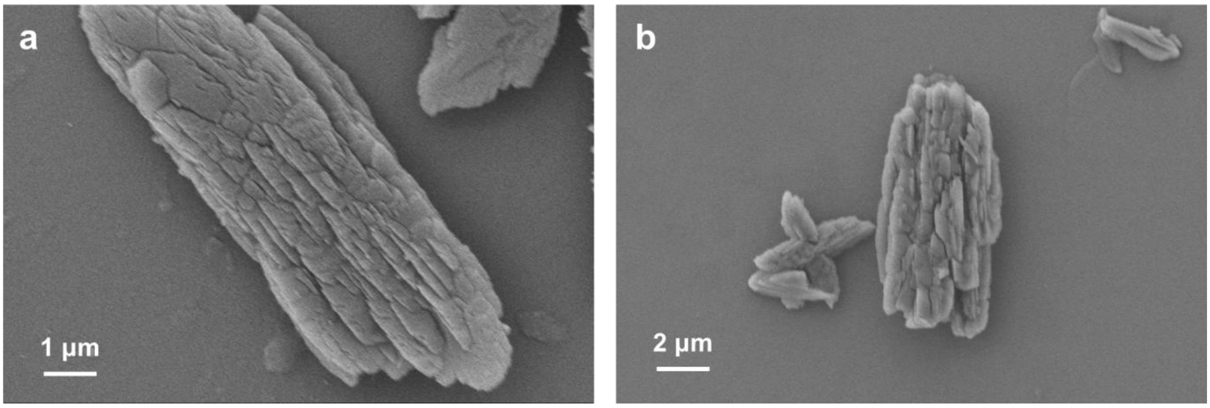

Figure S21. The SEM images of FDU-HOF-5 before and after the adsorption of DCP.

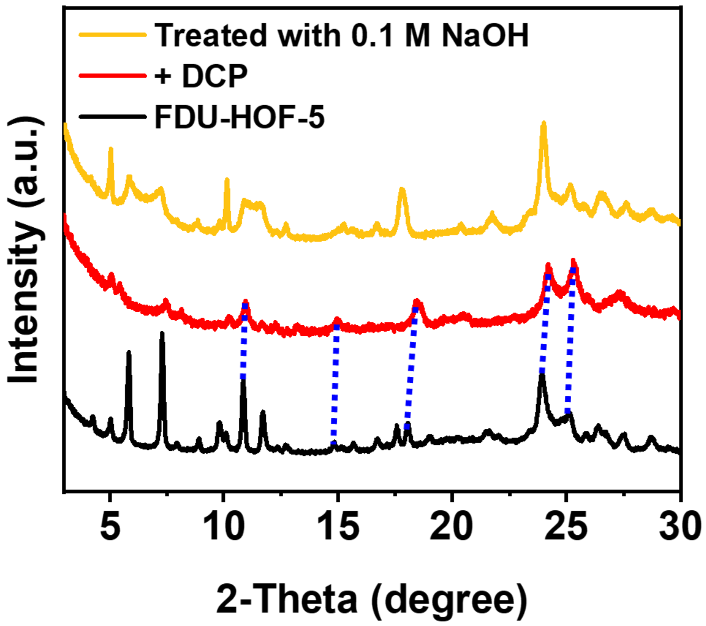

Figure S22. The PXRD spectra of FDU-HOF-5 after the adsorption of DCP and after treatment with 0.1 M NaOH.

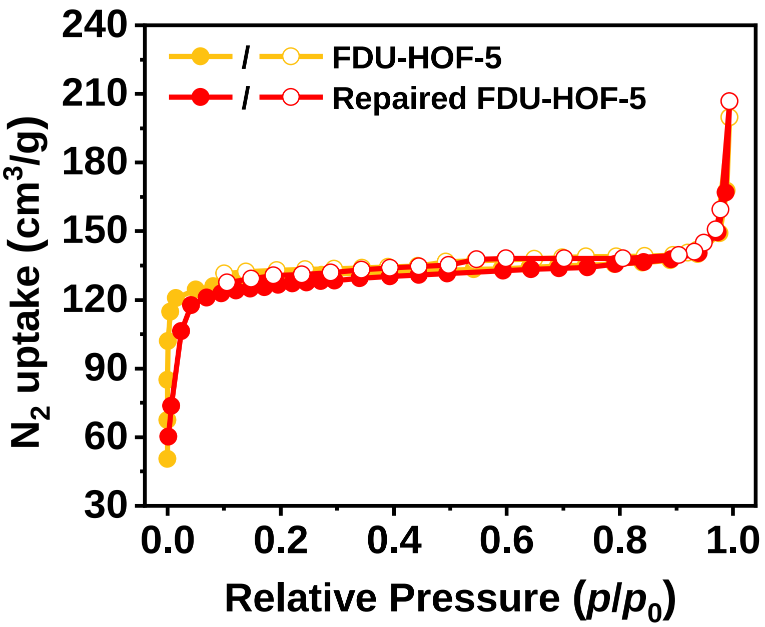

Figure S23. The 77 K N_2 adsorption isotherm of the repaired crystal.

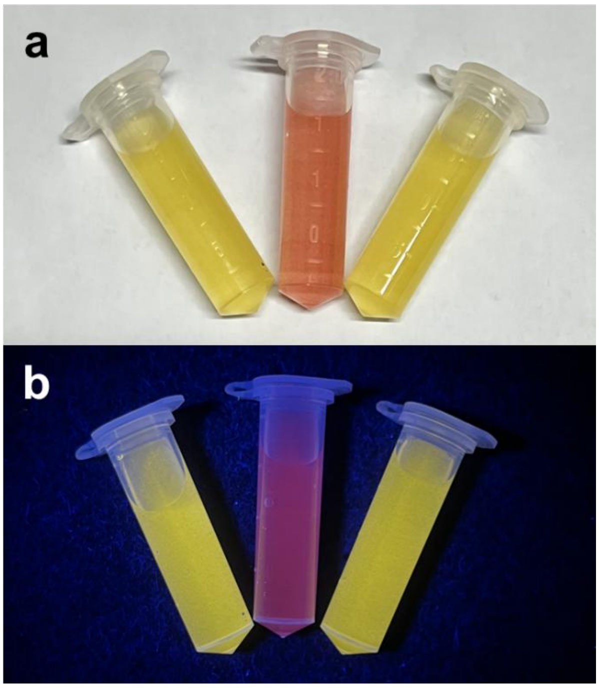

Figure S24. Photographs of FDU-HOF-5 (left), FDU-HOF-5⊃DCP (middle) and FDU-HOF-5⊃DCP treated with 0.1 M NaOH (right), (a) under daylight, (b) under UV (λ_{ex} = 365 nm) light.

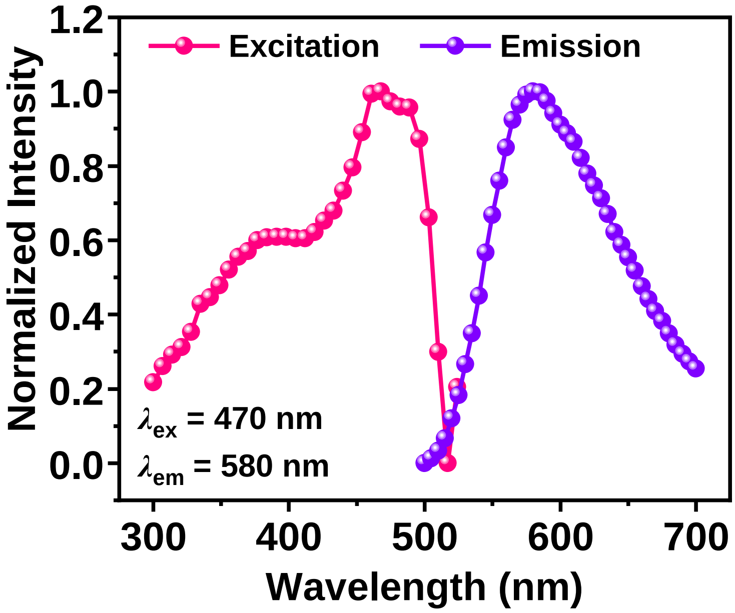

Figure S25. The fluorescence excitation and emission spectra of FDU-HOF-5.

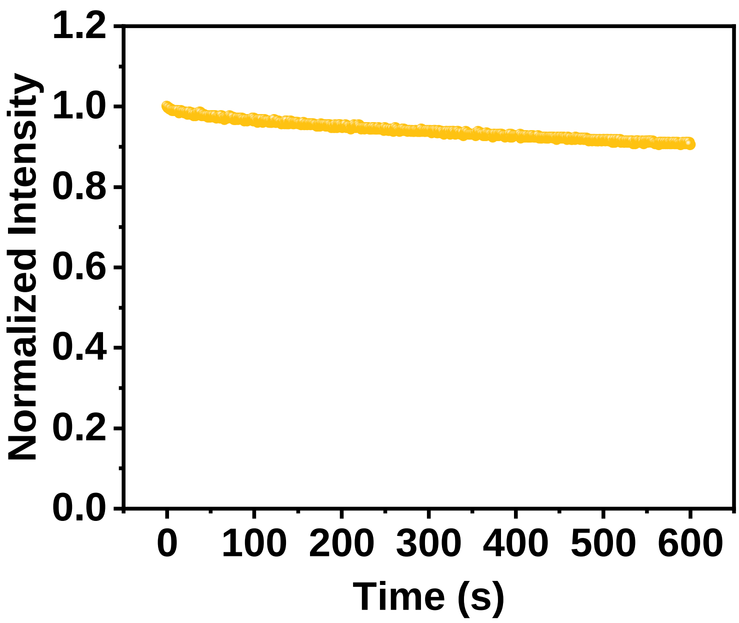

Figure S26. The image of the time-dependent variation of the luminescence intensity (λ_{em} = 580 nm) of FDU-HOF-5 under continuous irradiation with ultraviolet light (λ_{ex} = 365 nm).

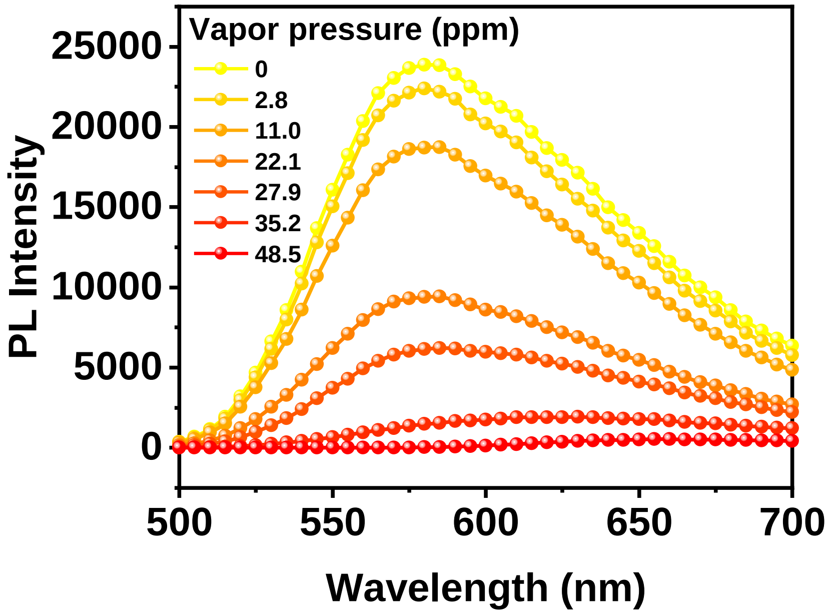

Figure S27. The fluorescence emission spectra changes of FDU-HOF-5 under the action of different concentrations of DCP.

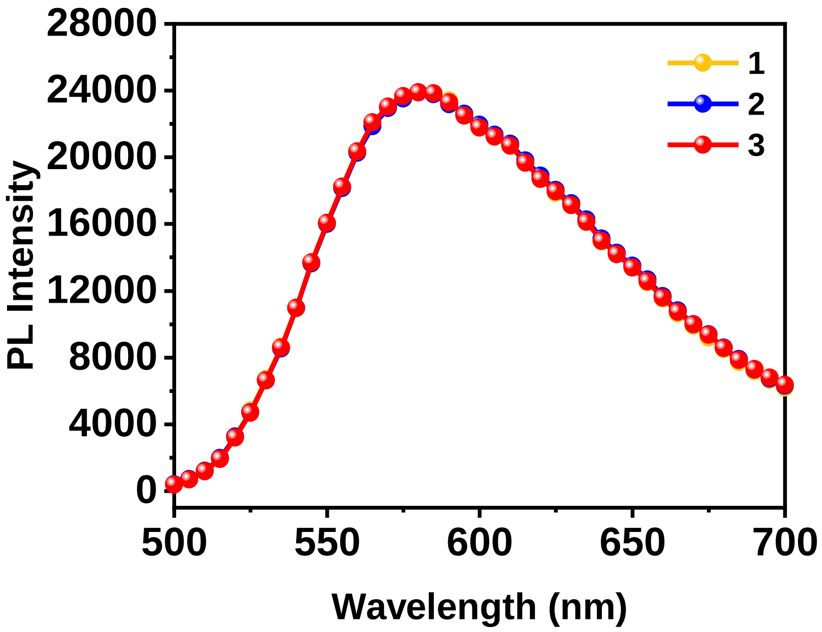

Figure S28. Three repeated fluorescent measurements of pristine FDU-HOF-5 solid.

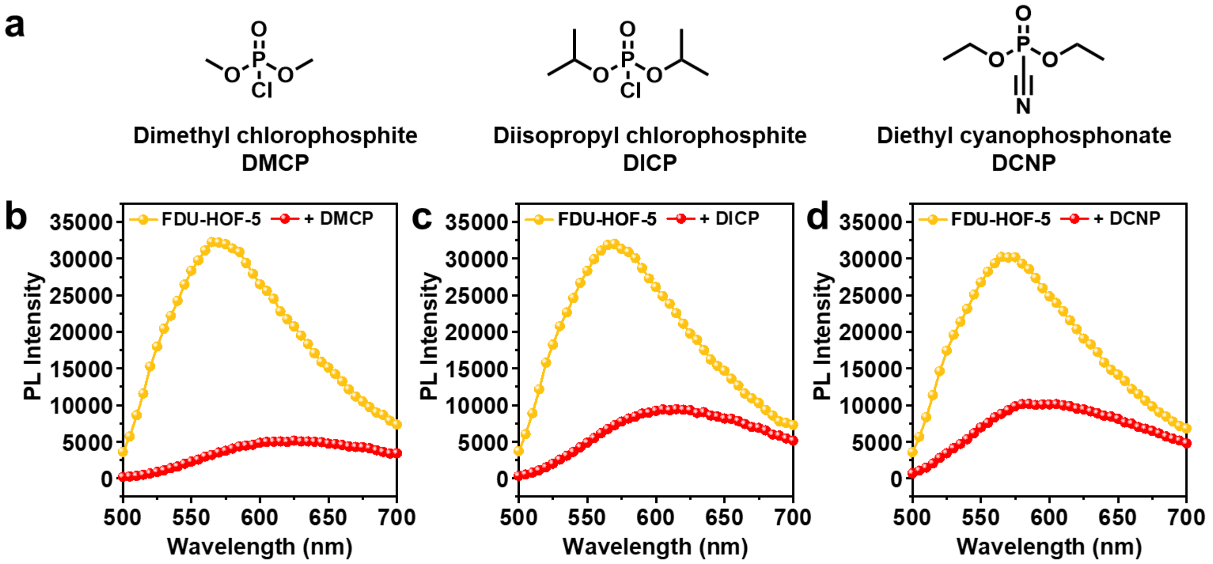

Figure S29. (a) The molecular structures of three other organophosphorus simulants, and their fluorescence spectra before and after interaction with FDU-HOF-5: (b) DMCP, (c) DICP, and (d) DCNP.

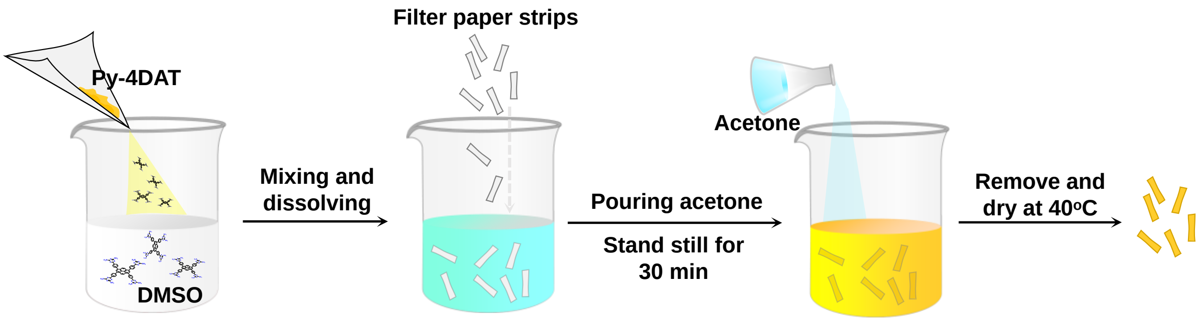

Figure S30. Flowchart of the preparation of the test strips for detecting DCP.

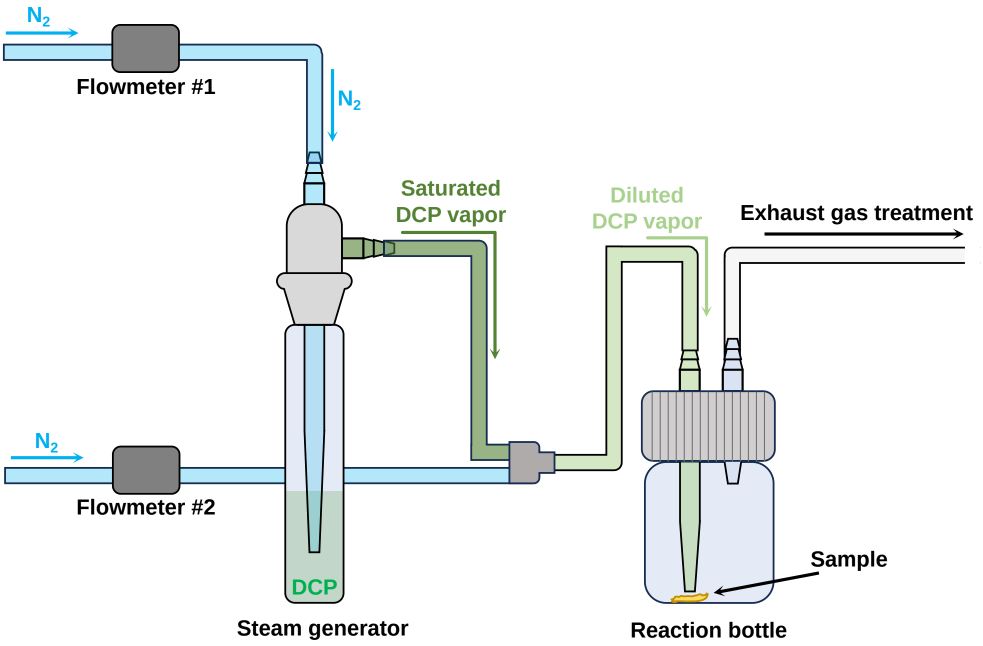

Figure S31. Schematic illustration of the fumigation device for FDU-HOF-5 in different vapor or vapor atmospheres of different concentrations. The transformation of vapor types is achieved by varying the liquid in the vapor generator; the vapor concentration is changed by adjusting the indications of the two gas flowmeters.

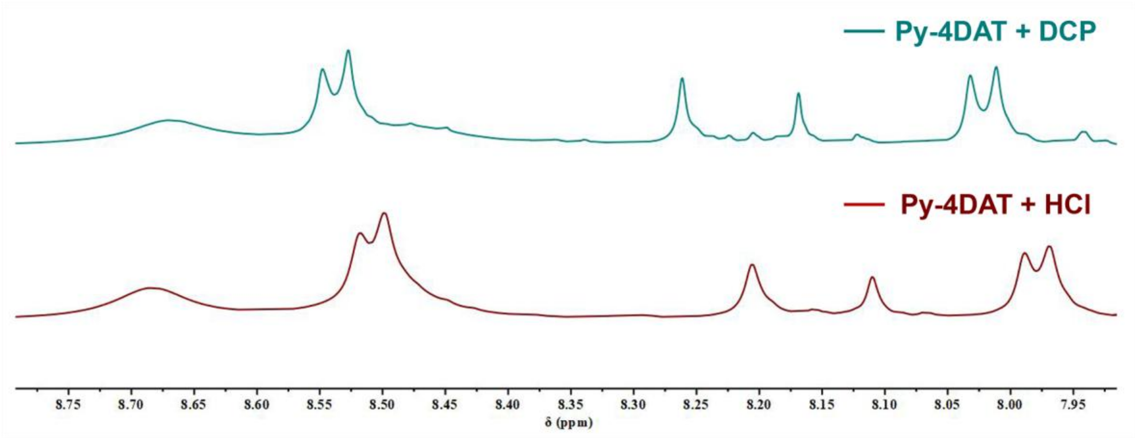

Figure S32. The ^{1}H NMR of Py-4DAT after its reactions with DCP and hydrochloric acid, respectively.

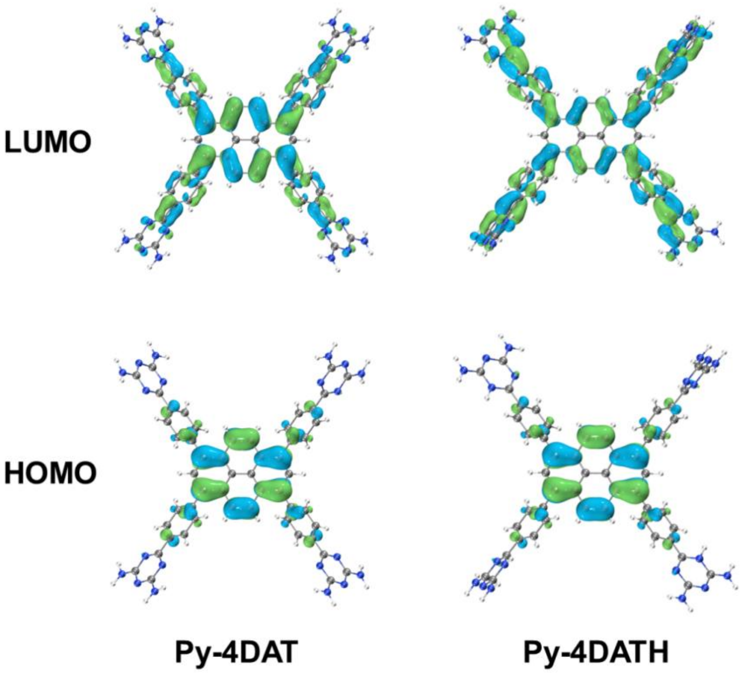

Figure S33. The spatial distribution diagrams of the HOMO and the LUMO of Py-4DAT and its protonated form Py-4DATH.

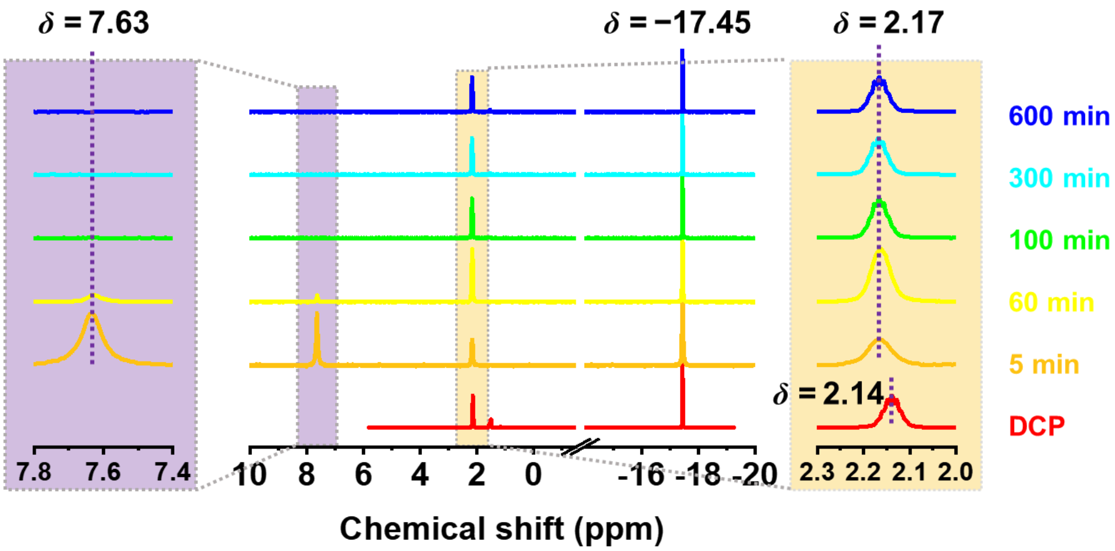

Figure S34. The ^{31}P NMR of the reaction of DCP with FDU-HOF-5 as a function of time.

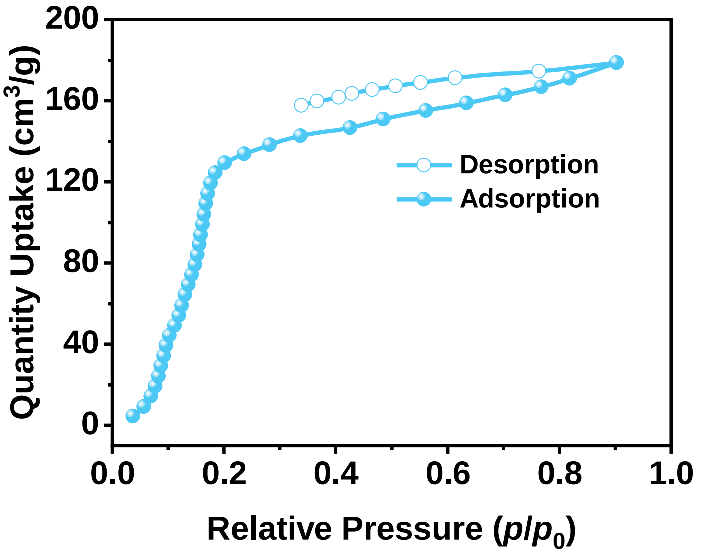

Figure S35. The water adsorption isotherm of FDU-HOF-5.

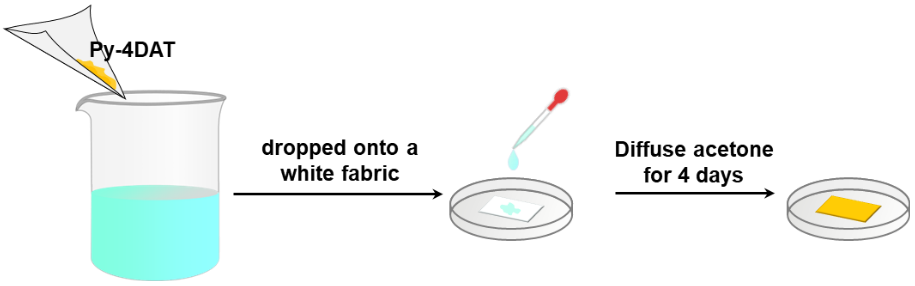

Figure S36. Standard procedure of FDU-HOF-5-loaded fabric.

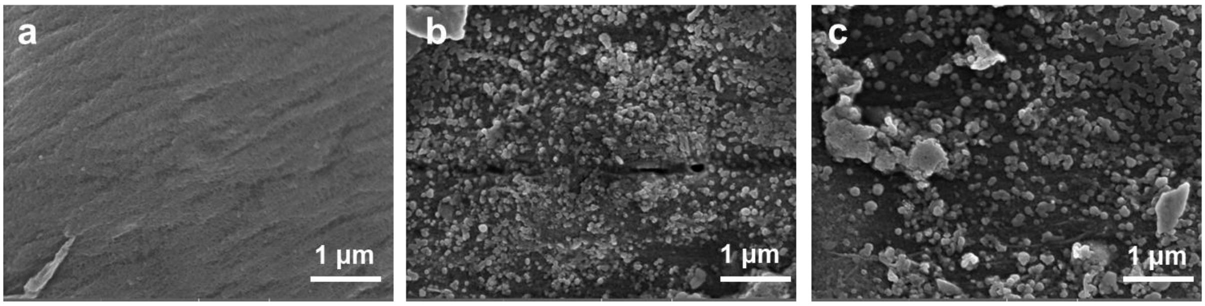

Figure S37. SEM images of (a) original fabric, (b) FDU-HOF-5-loaded fabric, and (c) FDU-HOF-5-loaded fabric after bending.

19. References.

[1] P. Li, Y. He, H.D. Arman; et al. "A Microporous Six-Fold Interpenetrated Hydrogen-Bonded Organic Framework for Highly Selective Separation of C_2H_4/C_2H_6," *Chemical Communications* (2014): 13081, doi:10.1039/c4cc05506c.

[2] H. Wang, B. Li, H. Wu; et al. "A Flexible Microporous Hydrogen-Bonded Organic Framework for Gas Sorption and Separation," *Journal of the American Chemical Society* (2015): 9963, doi:10.1021/jacs.5b05644.

[3] M.J. Frisch, G.W. Trucks, H.B. Schlegel; et al. *Gaussian 16*, Revision C.01; Gaussian, Inc.: Wallingford CT, 2019.

[4] T. Lu, F. Chen. "Multiwfn: A Multifunctional Wavefunction Analyzer," *Journal of Computational Chemistry* (2011): 580, doi:10.1002/jcc.22885.

[5] T. Lu. "A Comprehensive Electron Wavefunction Analysis Toolbox for Chemists, Multiwfn," *The Journal of Chemical Physics* (2024): 082503, doi:10.1063/5.0216272.

[6] Z. Liu, T. Lu, Q. Chen. "An sp-Hybridized All-Carboatomic Ring, Cyclo[18]carbon: Electronic Structure, Electronic Spectrum, and Optical Nonlinearity," *Carbon* (2020): 461, doi:10.1016/j.carbon.2020.05.023.

[7] J. Zhang, T. Lu. "Efficient Evaluation of Electrostatic Potential with Computerized Optimized Code," *Physical Chemistry Chemical Physics* (2021): 20323, doi:10.1039/d1cp02805g.

[8] W. Humphrey, A. Dalke, K. Schulten. "VMD: Visual Molecular Dynamics," *Journal of Molecular Graphics* (1996): 33, doi:10.1016/0263-7855(96)00018-5.

[9] *Materials Studio*, 7.0; Accelrys: San Diego, California (USA), 2019.

[10] G. S. Pawley. "Unit-Cell Refinement from Powder Diffraction Scans," *Journal of Applied Crystallography* (1981): 357, doi:10.1107/s0021889881009618.

[11] Y.-K. Lee. "Comparison of CIELAB ΔE^* and CIEDE2000 Color-Differences after Polymerization and Thermocycling of Resin Composites," *Dental Materials* (2005): 678, doi:10.1016/j.dental.2004.09.005.

[12] C.-C. Xing, C. Gao, X. Zhang; et al. "Amino-Triggered Emission in Biological Metal-Organic Frameworks for Nerve Agent Simulant Detection," *Analytical Chemistry* (2025): 10858, doi:10.1021/acs.analchem.5c01277.

[13] M. Ran, Y. Liu, L. Feng; et al. "Amino-Functionalized Nano-UiO-66 for the Detection of Nerve Agent Analogs," *ACS Applied Nano Materials* (2025): 8231, doi:10.1021/acsanm.5c00594.

[14] Y. Lei, N. Gao, P. Huang; et al. "UiO-66-NH_2 Initiated Cascade Reaction: Constructing a Ratiometric Fluorescence Sensor for Ultrasensitive Detection of Nerve Agent Simulant," *Analytica Chimica Acta* (2024): 342421, doi:10.1016/j.aca.2024.342421.

[15] Z. Shen, W. Li, W. Tang; et al. "Fluorophor Embedded MOFs Steering Gas Ultra-Recognition," *Advanced Functional Materials* (2024): 2401631, doi:10.1002/adfm.202401631.

[16] J. Pang, Y. Liu, C. Zhao; et al. "One-Step Coordinating POPD in H_3BTB-Sensitized EuMOF-Enabled Tunable Antenna Effects for Fluorescence Turn-On Sensing of Sarin Analogue Vapor," *ACS Applied Materials & Interfaces* (2025): 25722, doi:10.1021/acsami.5c04451.

[17] Y. Gong, Y. Guo, C. Qiu; et al. "Integrative Self-Assembly of Covalent Organic Frameworks and Fluorescent Molecules for Ultrasensitive Detection of a Nerve Agent Simulant," *Science China Materials* (2020): 1189, doi:10.1007/s40843-020-1517-8.

[18] C. C. Xing, H. R. He, J. Ma; et al. "Pore-Space-Partition Enabled Through-Space-Charge-Transfer in Metal-Organic Frameworks for Enhanced Sarin Simulant Recognition," *Advanced Functional Materials* (2025): e19089, doi:10.1002/adfm.202519089.

[19] S. Jindal, V. K. Maka, G. Anjum; et al. "Anthracene-Bisimidazole Tetraacid Linker-Based Metal-Organic Nanosheets for Turn-on Fluorescence Sensing of Nerve Agent Mimics," *ACS Applied Nano Materials* (2021): 449, doi:10.1021/acsanm.0c02727.

[20] X. Xu, M. Tian, Z. Lin; et al. "Cooperation Between Eu MOF and Glycerol for Luminescent Sensing of Nerve Agent Mimic Vapor," *Journal of Solid State Chemistry* (2022): 123114, doi:10.1016/j.jssc.2022.123114.

[21] Y. Lei, Y. Gao, Y. Xiao; et al. "Zirconium-Based Metal-Organic Framework Loaded Agarose Hydrogels for Fluorescence Turn-on Detection of Nerve Agent Simulant Vapor," *Analytical Methods* (2023): 5674, doi:10.1039/d3ay01539d.

[22] Q. Peng, C. Qiu, P. Huang; et al. "An Integrated Ratiometric Fluorescent Probe Based on the MOF-on-MOF Heterostructure for Sensitive Detection of Nerve Agent Simulant," *Talanta* (2026): 128528, doi:10.1016/j.talanta.2025.128528.

[23] S. Ghosh, R. Lipin, A. Ngoipala; et al. "Hf-Based MOF for Rapid and Selective Sensing of a Nerve Agent Simulant and an Aminophenol: Insights from Experiments and Theory," *Inorganic Chemistry* (2023): 14632, doi:10.1021/acs.inorgchem.3c01777.

[24] C. Lyu, C. Zhao, M. Wang; et al. "Exactly Restricting the Phenyl Ring Rotation in Metal-Organic Framework for Ultra-Sensitive and Specific Ratiometric Fluorescent Sensing of Sarin," *Aggregate* (2025): e70053, doi:10.1002/agt2.70053.

[25] Y. Lei, Y. Gao, Y. Xiao; et al. "Cu^{2+}-Functionalized Zr-MOF Triggers *O*-Phenylendiamine Oxidation for Ultrasensitive Ratiometric Fluorescence Detection of Nerve Agent Simulant," *Sensors and Actuators B: Chemical* (2023): 134553, doi:10.1016/j.snb.2023.134553.

[26] S.-J. Xiao, M.-Y. Yuan, Y.-D. Shi; et al. "Construction of Covalent Organic Framework Nanozymes with Photo-Enhanced Hydrolase Activities for Colorimetric Sensing of Organophosphorus Nerve Agents," *Analytica Chimica Acta* (2023): 341706, doi:10.1016/j.aca.2023.341706.

[27] S. Ma, M. Du, A. Shen; et al. "The Selective Identification of Nerve Agent and Mustard Gas Simulants Based on the Multi-Functionalized Luminescent Platform of Tb^{3+}@UiO-66-DPA," *Microporous and Mesoporous Materials* (2022): 112006, doi:10.1016/j.micromeso.2022.112006.
